# Supplementary material for: The genomic ancestry of the Scandinavian Battle Axe Culture people and their relation to the broader Corded Ware horizon
Source: Proc Biol Sci. 2019 Oct 9;286(1912):20191528. doi: 10.1098/rspb.2019.1528 (PMC6790770; doi:10.1098/rspb.2019.1528)
Supplement: Supplementary Text [file rspb20191528supp1.pdf]

The genomic ancestry of the Scandinavian Battle Axe Culture people and their relation to the Corded Ware horizon

Helena Malmström, Torsten Günther, Emma M Svensson, Anna Juras, Magdalena Fraser, Arielle R Munters, Łukasz Pospieszny, Mari Tõrv, Jonathan Lindström, Anders Götherström, Jan Storå, Mattias Jakobsson

Proceedings of the Royal Society B, DOI 10.1098/rspb

Electronic Supplementary Material

Archaeological background **2**
Archaeological samples **5**
Bergsgraven, Östergötland, Sweden 5
Obłaczkowo, Wielkopolska, Poland 5
Karlova, Tartu, Estonia 6
Öllsjö, Scania, Sweden 6
Rössberga, Västergötland, Sweden 7
Ajvide, Gotland, Sweden 7
Radiocarbon dating and stable isotope analyses **8**
Sample preparation **12**
Bioinformatic data processing **12**
Authentication **13**
Deamination patterns 13
Contamination estimates 16
Uniparental markers **16**
Mitochondrial DNA 16
Y-chromosomal DNA 18
Phenotypic traits **20**
Comparative genome data **22**
Population genetic analyses **27**
Principal Component analysis 27
Unsupervised admixture 27
f4 statistic and Admixture graphs 28
Kinship analyses 34
References **35**

Archaeological background

The Corded Ware Culture (CWC) complex is found over large areas of central and northern Europe in the 3rd Millennium BCE. The appearance of the culture marks several important transitions seen in the archaeological (material) record where the most visible manifestations are typically, most often, single inhumation burials, battle-axes, and cord-decorated pottery [e.g. (1–3)]. The CWC complex appears rather simultaneously in many areas surrounding the Baltic Sea, including present day Poland, Denmark, the southern parts of Sweden and Norway, as well as Lithuania, Latvia, Estonia and southwestern Finland (4–8). In Sweden the CWC is distributed up to Middle Sweden [e.g. (4,9)] but finds also occur further to the north along the Baltic coast (4,9–11). On the eastern side of the Baltic Sea the CWC dispersed to the north as far Estonia and the southwestern parts of Finland [e.g. (12)]. The complex was preceded by different cultural groups in different areas around the Baltic Sea and was contemporaneous with other cultural groups in many areas, such as the Pitted Ware Culture (PWC) in Sweden and the Comb Ceramic Culture (CCC) groups in Estonia (13).

The earliest dates of CWC manifestations in Sweden are from the southern regions and dating to c.3000 BCE (14), in southern Finland from c.2900 BCE (15) and in Estonia c.3000/2800 BCE [e.g. (8,16)]. In Latvia and Lithuania, the earliest dates fall roughly in the same time interval around 3000 BCE. The earliest dates in Poland also fall around 3000/2800 BCE (17–20). The latest dates in most areas fall around 2400/2000 BCE. There are a few dates slightly older and also younger than this interval but their reliability has been under scrutiny, e.g. the earliest previously reported dates in Finland (7,15). Thus, the cultural manifestations of the CWC dispersed over large geographical areas within a rather limited time span and persisted for almost a millennium.

In Sweden, the CWC complex has been labeled the (Swedish-Norwegian) Boat Axe or Battle Axe culture (BAC), and displays specific cultural features such as specific stone axes, pottery and formalized burial customs that are different from those in the preceding periods in the area. Stray finds of the typical battle axes are distributed on the southern parts of Scandinavia and along the Baltic coastal areas to the North, figure S1A-B (21). The BAC is the equivalent to the Single Grave Culture (SGC) in Denmark and the CWC on the continent. How the BAC/CWC complex was brought to Sweden and Denmark has been extensively debated (6,9,22–29). The debate has, as in many other areas, centered on the question whether the introduction was associated with a migration event or cultural diffusion. The Scandinavian BAC complex was first identified by Sophus Müller (30) who, typically for the time, saw the emergence of the culture as a result of migration. That became a long lived interpretation, e.g. upheld by Forssander (25) who compiled the first more comprehensive overview of the Swedish finds. In contrast to earlier interpretations, Malmer (9) described the appearance of BAC in Sweden as a result of an internal development, an interpretation that also later has found strong support [e.g. (4,31–34)]. However, opposite views favoring the migration hypothesis and mobility have been presented [e.g (26)]. On the eastern side of the Baltic Sea, interpretations linking the appearance of the CWC complex to migrations and demographic transitions have found, and continue to find, support (8,13,16,24,35). Some studies, however, have stressed the importance of local groups in the adoption of the CWC elements [(36), see discussion in (37)]. Genetic data of ancient individuals from the Baltic countries indicate that a demographic transition occurred in the Baltic Middle and Late Neolithic, probably in association with the appearance and dispersal of the CWC complex (38–40). This seems to be the case also in Poland (41,42).

In southern Sweden, the BAC is the chronological successor of the Funnel Beaker Culture (FBC) [e.g. (27)]. Further north, in Middle Sweden, the material manifestations of the FBC disappears c. 500 years prior to the appearance of the BAC material culture. Instead, the

material manifestations of the PWC emerges in Eastern Middle Sweden and also in the southern parts of Scandinavia, mainly in the coastal areas and on the Baltic Islands of Öland, Gotland and the Åland Islands [e.g. (27)]. The BAC is contemporaneous with the late phase of the PWC and is visible in large numbers of stray finds of battle/boat shaped axes, a few settlement remains and a few burials which most often are distributed more inland than PWC sites [e.g. (4,27)]. There were long distance contact routes along the coast, stretching at least from Scania in the South to Västerbotten in the North, and via the Åland archipelago to Finland in the East (21). In the northern part of the distribution area in Sweden, territorial clusters of finds (figure S1B), have been interpreted as possible tribal areas (21). On the eastern side of the Baltic Sea the CWC complex succeeded groups associated to the CCC complex, except in Estonia, where CCC co-existed with CWC until the beginning of the Baltic Bronze Age [e.g. (7,8,13,16)].

The BAC complex has been seen by some archaeologists as a continuation and transformation of the FBC (9,27,31). In southern Scandinavia, radiocarbon dates from BAC contexts predates those from Middle Sweden. According to interpretations by Malmer (9) the BAC manifestations dispersed northwards, through diffusion, where new elements were incorporated into the earlier FBC complex. However, the BAC complex differs from the preceding FBC complex in many important aspects, with a new burial tradition involving a transition from collective burials to flat earth graves containing one or occasionally a few individuals, and with individual grave goods. Malmer (28) considered the emergence of the BAC a result of a new social and/or religious organization, where the economy was based on farming and husbandry. The association between the BAC and FBC, and also between BAC and PWC, in Sweden has been discussed at great length [e.g. (27,31–33)]. The subsistence economy of the PWC and BAC people was different, where the former was characterized by foraging and especially the exploitation of marine resources (43,44). The BAC economy included animal husbandry and possibly farming. Recent studies of stable isotopes indicate that the diet of BAC individuals was based mainly on terrestrial proteins, thus, more closely resembling that of FBC individuals than PWC individuals (45).

Recent analyses of the BAC/CWC pottery technology have shown similarities between Middle Sweden and Finland, and it has even been suggested that potters moved to Sweden from Finland (34). Possibly, the BAC complex dispersed from there to Eastern Middle Sweden [e.g. (34), see also (7,46)]. Most commonly in earlier research, the unifying and similar cultural traits within the BAC/CWC complex have been in focus (2). The similarities in burial customs, pottery design and typology, as well as the typical battle-axes, to name a few, have caught the attention of researchers and, thus, the traits have been seen as indications of a society with common cultural and social practices [see e.g. (1,2,9,22,24) for reviews]. In more recent research more attention has been focused on regional patterns and traits, and also deviances in e.g. burial customs, which shed another light on the cultural complex [e.g. (1,3,4)]. For instance, while Malmer (9) considered Scania and Blekinge a core are, different from other areas in Sweden, Edenmo (4) among others, have stressed that there are notable differences in the material culture expressions in different parts of Middle Sweden. Furholt (1) called for more or less the same kind of caution when interpreting the unity and general similarity within the BAC/CWC complex based on new archaeological data. In a similar manner, Kristiansen *et al*. (3) recently called for a revision of the CWC in Europe in light of new genetic and linguistic research, and with a specific focus on the adaptations and interactions with people of the Yamnaya complex. However, Heyd (47) and Furholt (2), among others, have called for caution and highlighted a need for integration of fuller and wider archaeological perspectives in the interpretations of e.g. the transition between the Yamnaya and the CWC complexes. This (social) process is not well understood, even in light of the new genetic data. The analyses of individuals from the northern parts of the distribution area of the BAC/CWC have good potential to highlight these demographic processes.

The formation of the CWC complex in continental Europe appears to be associated to the emergence of the Yamnaya complex that appeared in the eastern parts of central Europe around 3000 BCE. The similarities in many cultural expressions between the CWC and Yamnaya complexes have been debated for long [e.g. (35)]. Archaeogenomic analyses have found evidence of a wave of migration into Europe from the Pontic–Caspian steppe, which also had an impact on the demographic development of the CWC complex in large areas of central and northern Europe (41,48,49). Earlier studies have shown that individuals from CWC contexts in Poland exhibit a genomic input from the eastern Yamnaya steppe pastoralist (41). This genetic “steppe” component, have been identified in varying amounts, also among CWC individuals in Estonia and Latvia (38–40). The steppe component has been observed in Scandinavian Middle and Late Neolithic individuals from Sweden (39,48), but their burial contexts are not fully understood. Interestingly, the genetic influence of the Yamnaya pastoralist decreases in western European CWC individuals, but there is also a marked variation between individuals (50).

For the present study we have chosen eleven individuals for archaeogenetic analyses; five individuals from BAC or CWC contexts in Sweden, Poland and Estonia; five individuals recovered from two FBC associated megalithic burial structures in Sweden, and one individual from a PWC context on the Island of Gotland, contemporaneous with the early BAC phase in Sweden. By inclusion of individuals from Sweden, Poland and Estonia and individuals buried in different funerary contexts, we have the potential to examine possible regional patterns in genetic variation and affinities, as well as admixture patterns.

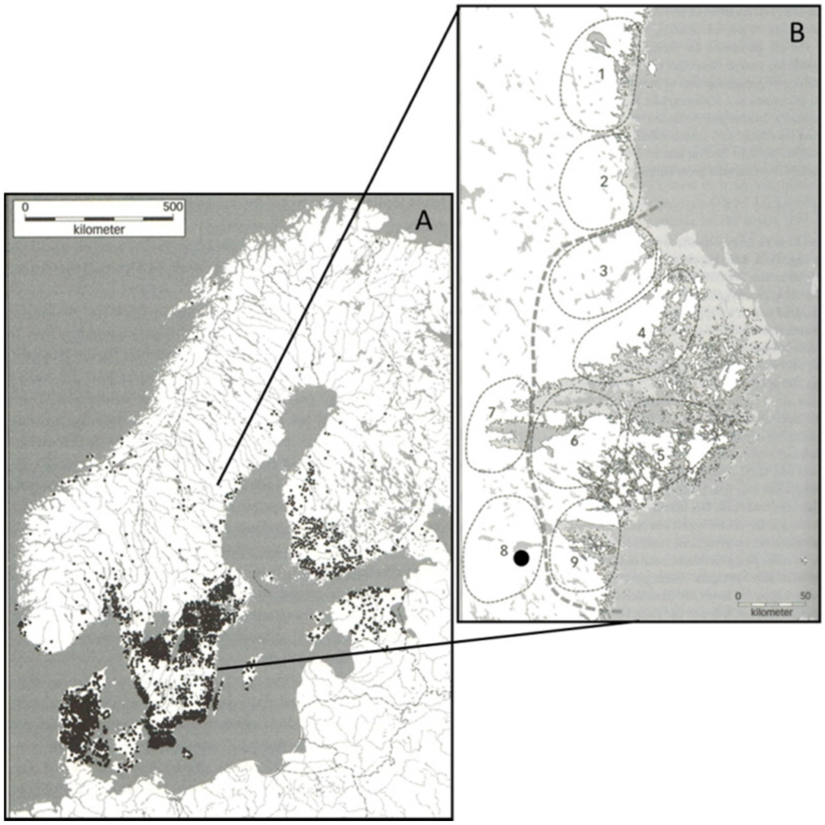

Figure S1. A) The distribution of BAC/CWC battle axes in Denmark, Sweden, Norway, Finland, and Estonia [map modified from (21)]. B) Hypothesized social territories in Eastern Middle Sweden during 2900-2400 BCE and location of Bergsgraven in Linköping [map modified from (21)].

Archaeological samples

Bergsgraven, Östergötland, Sweden

Bergsgraven (grave no. 185) in Linköping is a well-preserved triple grave attributed to the BAC. It is the northernmost BAC type of burial with preserved unburnt human remains in Sweden. This flat earth grave was excavated in the1950s and contained a male and a female buried in hocker position in an east-west direction (51,52). The male was buried on his right side and the female on her left side with their heads towards south/southeast. The grave contained a wealth of grave goods including a battle axe, zigzag ornamented pottery and among the oldest metal objects found in Sweden (two copper spirals) [see e.g. (9) for description of many of the finds]. Some of the objects were made out of material not from the area, such as scanian flint and amber. Animal remains include a complete dog buried behind the female, as well as bone implements of both wild species, e.g. a lower jaw of beaver, a larger implement of red deer antler, a needle of deer antler, and domestic species, as well as a burin of a metapodial made of sheep/goat. Of some interest is the similarity of the bone needle found next to the male which resembles one find in Estonia but also other finds in Sweden [see (53), see also (9,54)]. The third individual in the grave was not discovered until 1982, when some small bones close to the dog was shown to belong to an infant.

> ber1: An adult male, 25-30 years old at time of death, and approximately 177-180 cm tall. Two mandibular teeth (M1 dxt and M2 dxt) were sampled for DNA, stable isotope analyses and radiocarbon dating. This individual was dated to 2620-2470 cal BCE (95.4%) (table 1, table S1, Supplementary Section Radiocarbon dating and stable isotope analyses).

> ber2: A young female, 18-20 year, and approximately 162-162 cm tall. Two mandibular teeth (M1 sin and M2 sin) were sampled for DNA, stable isotope analyses and radiocarbon dating. This individual was dated to 2640-2480 cal BCE (95.4%) (table 1, table S1, Supplementary Section Radiocarbon dating and stable isotope analyses). She has previously been shown to belong to the N1a mitochondrial DNA (mtDNA) lineage (55).

Obłaczkowo, Wielkopolska, Poland

The CWC grave in Obłaczkowo was unearthed in the course of rescue excavations carried out between 2006 and 2008 by Henryk Klunder’s Pracownia Archeologiczno-Konserwatorska and supervised by Paweł Pawlak. The grave was located on a top of a small hill and no traces of a burial mound were found. It was rectangular in shape (2,4-2,5 x 1,9-2,15 m x 0,44 m) and oriented along the N-S axis. It contained skeletal remains of two adult individuals and one child, all buried in contracted position on a side. The grave offerings included a battle-axe and decorated antler plates (56). The grave belongs to the oldest stage of a small regional CWC group, covering the regions of Wielkopolska and Kujawy and known for ca. 40 burials (18).

> poz44: An almost complete although fragmented skeleton of a child (feature E8-A*). AMS radiocarbon dated to 2870-2580 cal BCE (95.4%) (table 1, table S1, Supplementary Section Radiocarbon dating and stable isotope analyses). Two mandibular teeth (I2 and C) and the petrous portion of the temporal bone were sampled for DNA. *We note that RISE1 (48) is also described as the individual from Obłaczkowo feature E8-A. However, their genetic results differ from ours. They present this individual as a molecularly determined male that belongs to Y-chromosomal haplogroup (hg) R1b and to mtDNA hg K1b1a1 (48) while our results show this individual to be female, carrying a mtDNA hg U3a’c profile (table 1, table

S4). We have backtracked all our handling of this sample and are therefore sure that we present data belonging to the child from Obłaczkowo feature E8-A.

poz81: An incomplete skeleton of an adult (feature E8-B). AMS radiocarbon dated to 2880-2630 cal BCE (95.4%) (table 1, tableS1, Supplementary Section Radiocarbon dating and stable isotope analyses). A phalange and the petrous portion of the temporal bone were sampled for DNA.

Karlova, Tartu, Estonia

The Karlova burial (National Registry of Cultural Monuments no. 12977) in southeastern Estonia was found during construction work in 1910 and was retrieved without proper excavation (57). The skeleton was buried in a supine position and oriented in a north to south direction. According to Hausmann (57), it belonged to an adult male. The general interpretation of the grave is that it belongs to the CWC complex, as it contained a battle axe made of diorite. The particular type of battle axe, the Karlova-type, was named after this burial and is thought to have been developed locally in western Estonia although having influences from the Finnish CWC (58). The second item in the grave was a Pyheensilta or Nylev type arrowhead made from phyllite. These are distributed in Finland, Norway, the Kola Peninsula and as single finds in the Baltic countries and date to the Late Neolithic, and are also associated with the CCC [see (16,58) and references therein].

kar1: An incomplete skeleton estimated, based on morphology, to belong to an 18-20 year old male with a height of approximately 170 cm (57). Note however, that we find the molecular sex of this individual to be female (table 1). The mandible has previously been AMS radiocarbon dated to 2440-2140 cal BCE (95.4%) (Poz-15499: 3805±35 BP, table 1) (16,59). One maxillary tooth (M3 sin) was sampled for DNA.

Öllsjö, Scania, Sweden

Ölljsö 7 (LUHM 28775) is a Scandinavian Middle Neolithic passage grave from Skepparslöv parish in Scania, Sweden. The tomb was excavated in 1943 (unpublished report by S. Hommerberg, available in Lund University Historical Museum report archive) and it was noted that the passage grave was somewhat destroyed and converted into a Stone cist during the Scandinavian Late Neolithic. Artefacts associated to the FBC (e.g. 'megalithic' pot sherds, amber beads, a bone necklace and flint implements) and to the Late Neolithic (e.g. pot sherds, a bronze disc, a bone pin, a flint dagger, and a flint spear head) were found, as well as a clay pot associated to the BAC (60). The human remains were partly commingled and belonged to at least 14 individuals, as 14 mandibles was discovered together with bones and horns from red deer, roe deer and wolf. Age estimations from tooth wear patterns according to (61,62) were performed for this study by Therése Hedlund and showed that the majority of the individuals were young (five individuals were 5-8 years, two were 11-15 years , four were 17-25 years, three were 25-35 years, one was 35-45 years and one ≥ 45 years of age). Morphological sex estimations were performed in accordance with Buikstra and Ubelaker (63) and one individual was identified as female, one as male, three as likely females, one as likely male, while ten individuals could not be morphologically sex determined (six of these latter individuals were children). No radiocarbon dates were available for these individuals prior to our investigations.

oll007: Commingled human remains. A mandibular tooth (M3 dxt) was sampled for DNA. According to tooth wear, this individual was 45+ years at the time of death. This individual was dated to 2860-2500 cal BCE (95.4%), and is therefore contemporaneous with the BAC time period and does not belong to the primary FBC

burial phase in the tomb (table 1, table S1, Supplementary Section Radiocarbon dating and stable isotope analyses).

oll009: Commingled human remains. A mandibular tooth (M1 sin) was sampled for DNA. Tooth wear indicates that this individual was 17-25 years at the time of death. This individual was dated to 1930-1750 BCE (95.4%) and thus belongs to the Scandinavian Late Neolithic (table 1, table S1, Supplementary Section Radiocarbon dating and stable isotope analyses).

oll010: Commingled human remains. A mandibular tooth (M3 sin) was sampled for DNA. This individual was 35-45 years old according to the tooth wear. The mandibula was radiocarbon dated to 1880-1660 BCE (95.4%) and, as with oll009, this was a later burial within the tomb from the Scandinavian Late Neolithic (table 1, table S1, Supplementary Section Radiocarbon dating and stable isotope analyses).

Rössberga, Västergötland, Sweden

Rössberga (RAÄ Valtorp 2:1) is a passage grave associated with the FBC in Västergötland in mid-central Sweden. It is situated in the Falbygden area comprising a dense concentration of about 255 megalithic tombs (64). The tomb was fully excavated in 1962 (65–67) and it has been estimated that at least 128 adult individuals were buried there (68). Radiocarbon dates of human remains vary between c. 3400 and 2500 cal BCE with a concentration of dates prior to 3000 BCE (66,69). Two individuals were incorporated in the present study (ros003 and ros005). Both belong to the primary FBC use of the tomb, but show differences in their radiocarbon dates (table 1, table S1). Previous studies show that the megalithic tomb was also used for burials during the Scandinavian Late Neolithic/Early Bronze Age times (67). Another study, of stable isotopes of individuals from the FBC burial phase, indicated rather homogeneous terrestrial dietary patterns ($\delta^{13}C$ and $\delta^{15}N$) and also low levels of mobility ($\delta^{34}S$) (69).

ros003: Commingled human remains. A mandibular fragment with one tooth (M dxt). The tooth was sampled for DNA and was radiocarbon dated to 3330-2930 cal BCE (95.4%) (table 1, table S1, Supplementary Section Radiocarbon dating and stable isotope analyses).

ros005: Commingled human remains. A mandible with one tooth (M2 dxt). The tooth was sampled for DNA and was radiocarbon dated to 3090-2920 cal BCE (95.4%) (table 1, table S1, Supplementary Section Radiocarbon dating and stable isotope analyses).

Ajvide, Gotland, Sweden

The Ajvide site (RAÄ Eksta 171:1), in Eksta parish on Gotland, Sweden has a burial ground with 85 excavated flat earth graves (with 89 individuals) associated with the Scandinavian Middle Neolithic PWC. The site was discovered in 1922 and excavations were conducted in 1923, 1958 and between 1983 and 2009 (70–75). The majority of buried individuals were placed in single graves and on their back in a supine position. Examples of grave goods are pit-decorated pottery, fish hooks, harpoons, stone and flint axes and adzes as well as faunal remains from fish, seal and feral pigs/boar, reflecting their hunting/gathering lifestyle. Radiocarbon dates indicate an activity range between 3200-2300 cal BCE (70,76,77). Some of the buried individuals have been analyzed previously using archaeogenetic methods (55,78–81).

ajv54: This individual was buried in an east-west oriented burial pit (70). The skeleton was morphologically estimated to be a 25-35 years old male with a height of 165-175 cm (82). Two teeth (a mandibular C sin and maxillary M2 sin) was used for DNA extraction and the M2 was radiocarbon dated to 2900-2680 cal BCE (95.4%) (table 1, table S1, Supplementary Section Radiocarbon dating and stable isotope analyses).

Radiocarbon dating and stable isotope analyses

For the present study, bone samples from eight individuals were submitted to Accelerator Mass Spectrometry (AMS) radiocarbon dating and Isotope Ratio Mass Spectrometry (IRMS) analyses for carbon and nitrogen isotope values at Beta Analytic Inc. (Miami, Florida) and bone samples from two individuals were AMS radiocarbon dated (through funding to Łukasz Pospieszny from the Ministry of Science and Higher Education of the Republic of Poland, Research Grant n. N N109 287 137) at ^{14}CHRONO Centre for Climate, the Environment, and Chronology at the Queen's University in Belfast and at Poznań Radiocarbon Laboratory in Poznań (table S1). The radiocarbon values, including values from a previously dated kar1 individual (16,59), were calibrated using Oxcal online software version 4.2.4 (83), based on the IntCal13 atmospheric curve and all dating results were rounded to the nearest tenth value (84) (table 1, figure S2). For ajv54, a mean offset of 70 ± 40 (43), was used for the marine reservoir age correction (figure S2).

The earliest dated individual from Sweden is one of the Rössberga individuals (ros003), which according to the radiocarbon date, belongs to the primary FBC burial phase of the megalith. The dating of the other individual from the same megalith (ros005) indicates that this individual was buried shortly after or towards the end of the FBC phase, which lasted on the Swedish mainland up to c. 2800 BCE, but slightly longer on the Island of Gotland (85–87). The dates of four of the individuals (ber1, ber2, poz44, poz81), from the typical BAC/CWC contexts from Bergsgraven in Sweden and Obłaczkowo in Poland all group between 2800-2500 cal BCE (95.4%) while the CWC burial at Karlova (16,59) in Estonia (kar1) is slightly younger. The individual from Ajvide (aj054) is roughly contemporaneous with the four BAC/CWC burials, as is the earliest individual (oll007) from the Öllsjö megalithic tomb. The remaining two individuals from Öllsjö (oll009 and oll010) had later dates, and fall within the Scandinavian Late Neolithic/Early Bronze Age. Stable isotope analyses ($\delta^{13}C$ and $\delta^{15}N$) of the individuals from Sweden show a limited variation, similar to earlier studied BAC individuals from Scania [data from (45)] (table S1, figure S3). The values indicate that the diet mainly incorporated proteins of terrestrial sources. Fornander (45) also demonstrated differences between the individuals in Scania, where a few had incorporated marine proteins. Larger variations in dietary patterns have been observed between individuals, and between different regions in Germany (88) and southern Poland (19). The isotopic signal of the PWC associated Ajvide individual (ajv54), deviate markedly from the other values, by an inclusion of mainly marine proteins in the diet. This is in line with earlier studies of PWC individuals [e.g. (43,89)].

Table S1. Conventional radiocarbon ages (i.e. uncalibrated BP values) and calibrated dates (BCE) of ten AMS dated samples in this study and IRMS stable carbon and nitrogen isotope data for eight of the samples.

Sample	C14 Lab ID	C14 age (BP)	Calibrated date (BCE, 95% CI)	$\delta^{13}C$ (o/oo)	$\delta^{15}N$ (o/oo)
ber1	Beta - 423305	4020 ± 30	2620-2470	-21.8	9.2
ber2	Beta - 423306	4030 ± 30	2640-2480	-20.3	10.2
oll007	Beta - 417630	4090 ± 30	2860-2500	-20.6	8.6
oll009	Beta - 417631	3520 ± 30	1930-1750	-19.6	9.5
oll010	Beta - 417632	3440 ± 30	1880-1660	-20.1	10.8
ros003	Beta - 423311	4440 ± 30	3330-2930	-21.6	9.0
ros005	Beta - 423313	4390 ± 30	3090-2920	-20.9	10.0
ajv54	Beta - 423308	4280 ± 30	2900-2680	-14.5	17.4
poz44	UBA – 16633	4117 ± 28	2870-2580	nd	nd
poz81	Poz - 36250	4160 ± 35	2880-2630	nd	nd

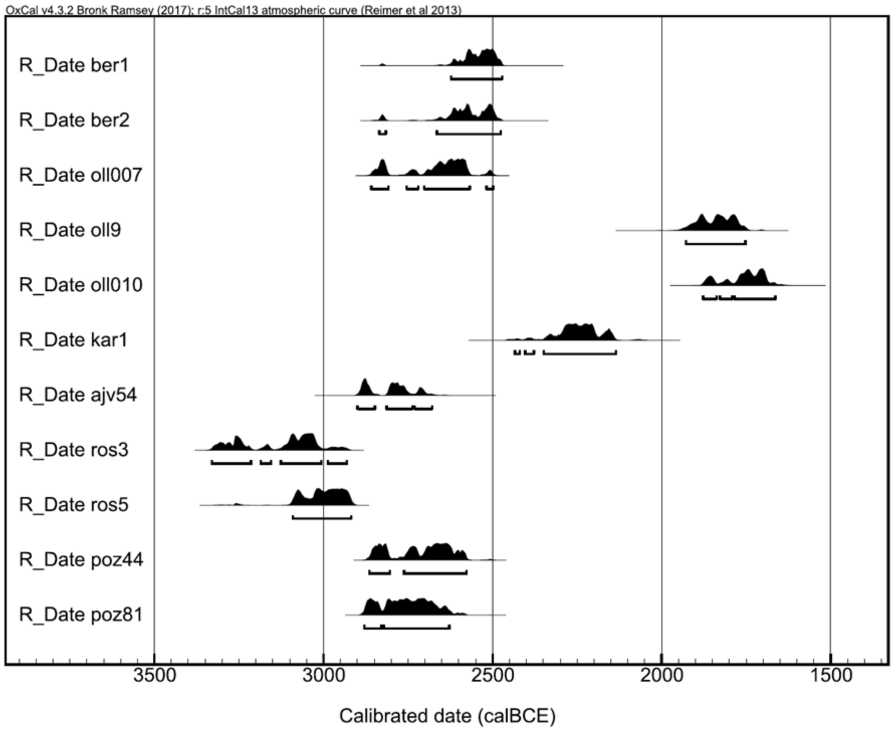

Figure S2. Calibrated radiocarbon dates (95.4% CI) for all eleven individuals in this study. The ajv54 sample was adjusted for marine reservoir effect by a mean offset of 70±40 (43).

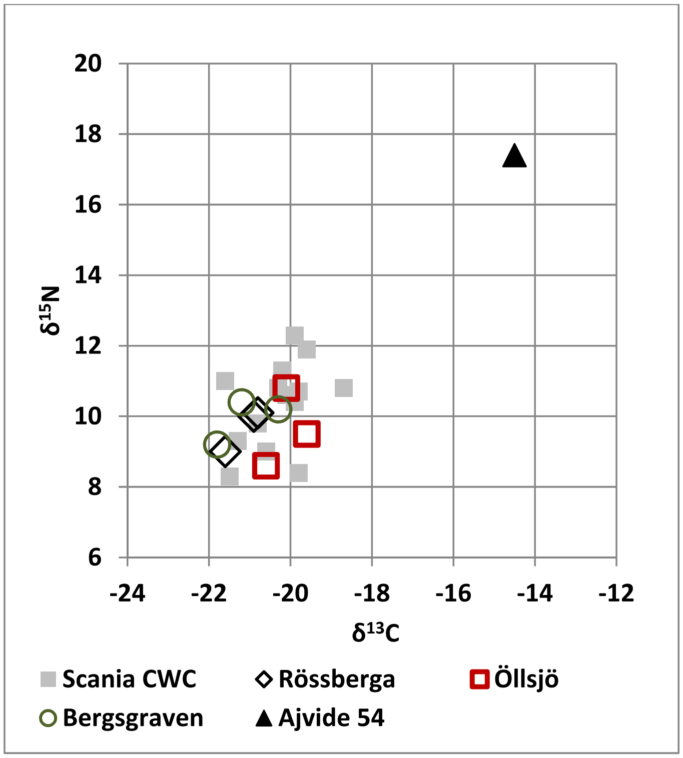

Figure S3. Dietary stable isotope values (δ13C and δ15N) from the eight individuals from Sweden analyzed in this study plotted together with BAC/CWC individuals from Scania (45).

Tooth enamel from the first and second molars of ber1, ber2, and from a faunal sample from the same burial (a dog), were subjected to strontium isotope analysis ($^{87}Sr/^{86}Sr$) at NERC, Isotope Geosciences Laboratory, Nottingham, UK (Professor Jane Evans) using their standard procedures as described in (85). The results indicate that the male and the female from Bergsgraven spent their childhood (here referring to the ages of c. up to 3 years (M1) and 8 years (M2)) in different geographic areas (table S2, figure S4). Both individuals seem to have stayed in their respective area at least for the first eight years of their lives. The Sr ratios for the dog is different from both individuals. Even if the two individuals spent their childhood in different places, it is difficult to assess where this would have been. The bedrock in Östergötland is varied and includes Precambrian rock (of granitoids and rhyolites) of different age but also smaller areas with younger Phanerozoic sedimentary rocks [see e.g. (90)]. The bioavailable strontium in Östergötland is very varied and soil samples exhibit values between 0.71623 and 0.74353 (90), i.e. covering the range of variation for both Bergsgraven individuals and as well as the dog. It is, thus, not possible to more closely evaluate the pattern of life history mobility. In a previous study of BAC individuals from Scania, low levels of mobility was noted on the basis of stable Sulphur isotope ($\delta^{33}S$) values (45).Variable levels of mobility have further been noted between different areas, or sites in Germany (88) and southern Poland (19).

Table S2. Strontium isotope values for mandibular teeth from the two individuals from Bergsgraven and from a faunal sample from the same burial.

Individual	Sample ID	Tooth	ppm	$^{87}Sr/^{86}Sr$
Bergsgraven 1, male	Ber1M-b2	M1 dx inf	94.0	0.73485
Bergsgraven 1, male	Ber1M-b1	M2 DX int	62.7	0.73391
Bergsgraven 2, female	Ber2F-b1	M1 sin inf	140.9	0.72350
Bergsgraven 2, female	Ber2F-b2	M2 sin inf	202.0	0.72529
Bergsgraven, dog	Ber3-b1	M2 sin inf	519.8	0.71743
Bergsgraven, dog	Ber3-b2	P3 sin inf	519.8	0.71611

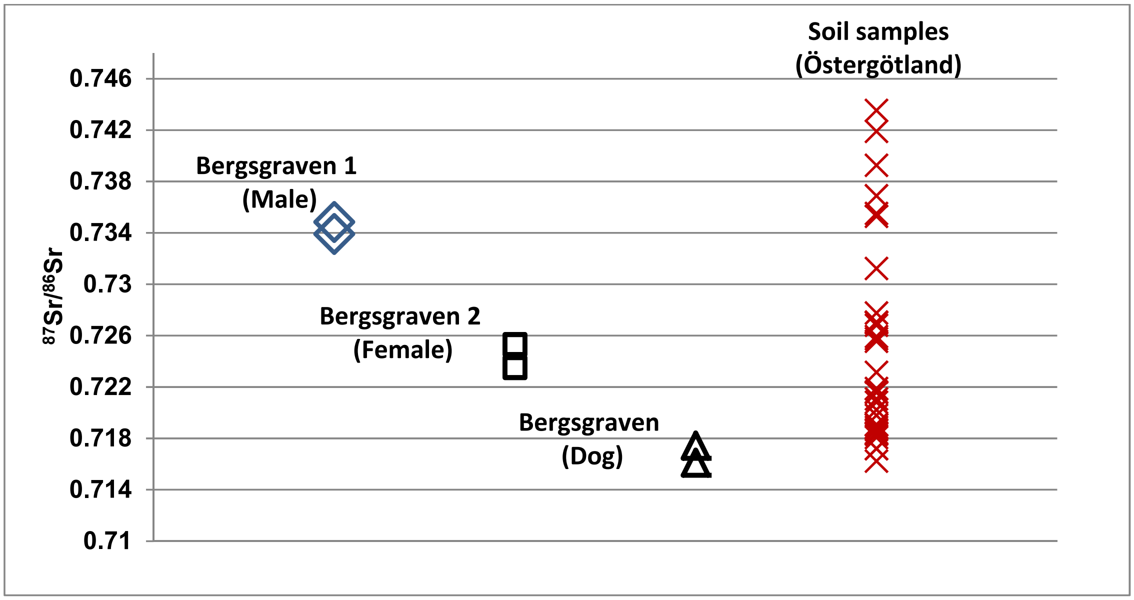

Figure S4. Strontium isotope values of the first and second molars from the male, female and dog from Bergsgraven plotted together with values from soil samples from Östergötland (90).

Sample preparation

We generated genome data from eleven ancient individuals using dedicated ancient DNA facilities [e.g. (91)] in Sweden (Human Evolution, Uppsala University; ber1, ber2, ros003, ros005, oll007, oll009 and oll010 and Campus Gotland, Uppsala University; kar1) and Poland (Department of Human Evolutionary Biology, Adam Mickiewicz University in Poznan; poz44 and poz81). Tooth samples were wiped with a 1% Sodium Hypoclorite solution and all samples were UV irradiated at 254nm for 6J/cm2. About 1 mm of the root/bone surface was removed before drilling out 30-100 mg of bone powder. DNA was extracted using silica-based methods (92) with modifications as in Malmström *et al*. (93) or Dabney *et al*. (94) and eluted in 40-110 µl of Elution Buffer (Qiagen). Between one and three extractions were made for each individual and typically one negative extraction control accompanied every six to eight of the ancient samples.

For each individual, between two and nine Illumina multiplex DNA libraries were prepared, together with one negative library control for every 6-8 ancient DNA libraries. The majority of libraries were prepared using blunt-end ligation with P5 and P7 adapters and indexes described in (95), without the shearing step as ancient DNA is already fragmented, and with amplification for 12-16 cycles using IS4 and index primers from Meyer and Kircher (95) and purification and quantification as in (96) or (78). Some blunt-end libraries were prepared as in (80) using using NEBNext® 20 DNA Library Prep Master Mix Set for 454, or using separate reagents, coupled with P5 and P7 adapters and indexes (95). A few libraries were prepared either as in (81), with T/A-ligation (NEBNext Quick DNA Library Prep Master Mix Set for 454, New England Biolabs), inPE adaptors and Illumina indexes (Illlumina Multiplexing Sample Preparation Oligonucleotide Kit, Illumina) or as in (97) to generate single-strand libraries. Additionally, some libraries (one blunt-end library from ber1, ber2 and ajv54 and two blunt-end libraries from oll009 and oll010) were enriched using Mybait Human Whole Genome Capture Kit with “Caucasian baits” (both from MYcroarray) following the manufacturer´s instructions (Mybaits manual version 2.3.1). Negative controls did not yield any DNA and were therefore not sequenced. In one case, however, we used a dog sample as negative control for human DNA, and libraries from this specimen were sequenced. The DNA libraries were sequenced at the SciLife Sequencing Centre in Uppsala with either Illumina HiSeq XTen and paired end 150 bp chemistry, HiSeq 2500 with paired end 125 bp chemistry, or HiSeq2000 with paired end 100 bp chemistry.

Bioinformatics data processing

The paired-end sequencing reads were merged and trimmed using MergeReadsFastQ_cc.py (98) into a joint fragment if they overlapped by at least 11 base pairs. They were then mapped as single ended reads to the 1000 genomes variant of the hg19 reference using bwa aln (99). We employed the non-default parameters -l 16500 -n 0.01 -o 2 as in (96). All sequencing runs of the same library, including Whole Genome Captured blunt-end libraries, were merged into one bam file with samtools before duplicate removal. The proportion of human reads deriving from shotgun sequenced Whole Genome Capture data before duplicate filtering was relatively low for the BAC individuals ber1 (2.2%) and ber2 (8.9%) and for the PWC individual ajv54 (14.1%) and higher for the Scandinavian Late Neolithic individuals oll009 (69.9%) and oll010 (89.0%). Reads mapped to identical start and end positions were considered PCR duplicates and merged using a modified version of FilterUniqSAMCons_cc.py which ensured random sampling of bases at mismatches between the duplicates. All libraries for a single individual were then merged into one bam file using samtools. Finally, reads with more than 10% mismatches to the reference genome and fragments shorter than 35bp were excluded from the data. Biological sex of the individuals was inferred using reads mapping to the X and Y chromosome (100). All data was used for downstream analyses.

Authentication

Deamination patterns

All sequenced samples showed a fragmentation and deamination pattern as expected for authentic ancient DNA (Figure S5) (101).

ajv54

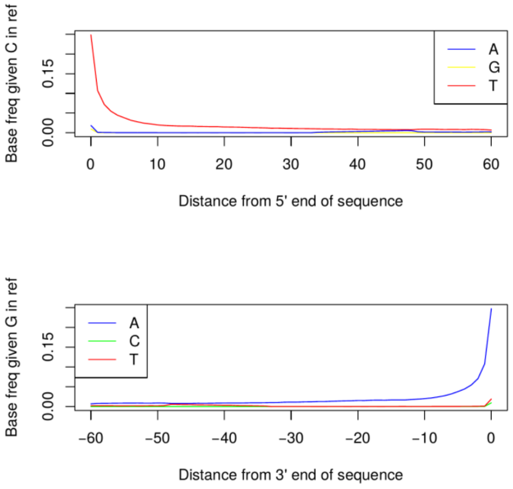

ber1

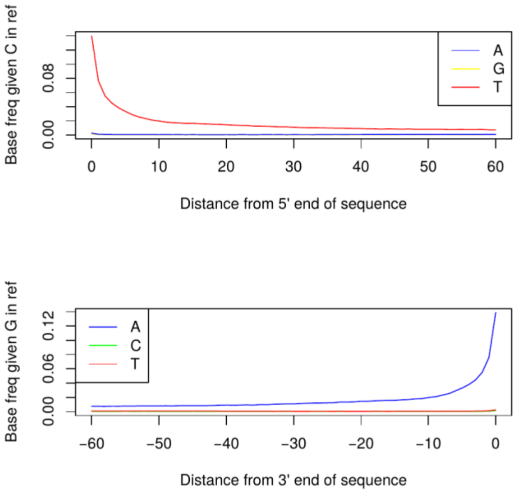

ber2

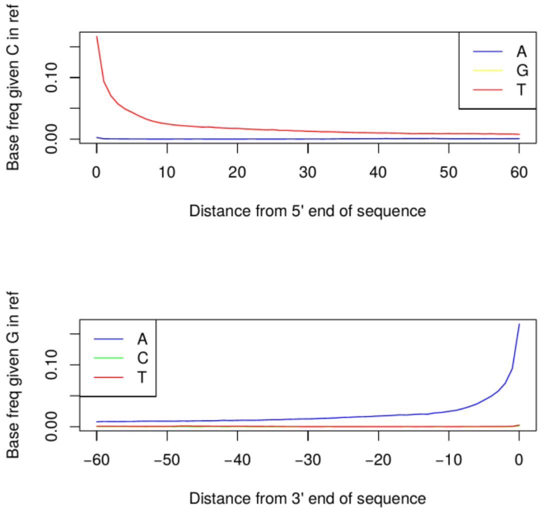

kar1

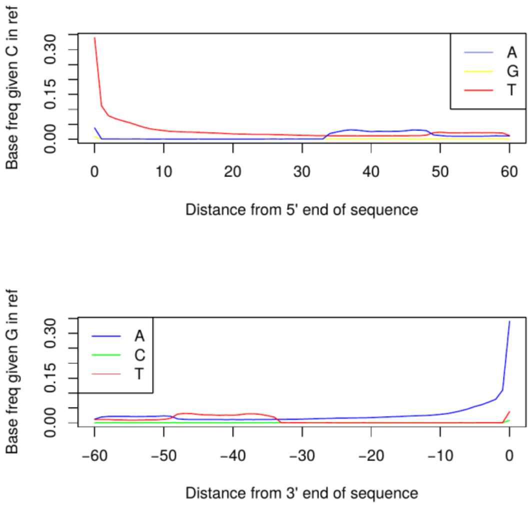

oll007

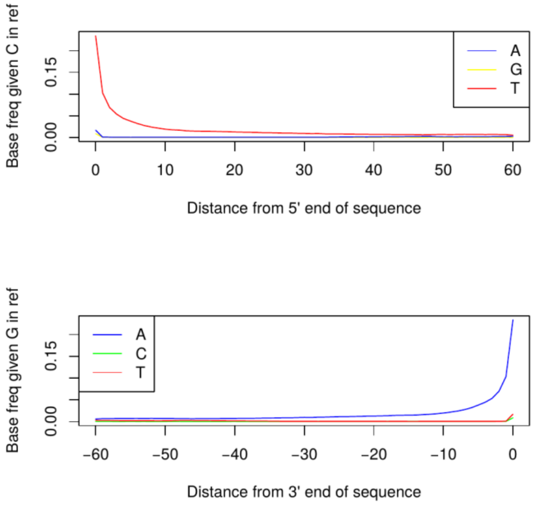

oll009

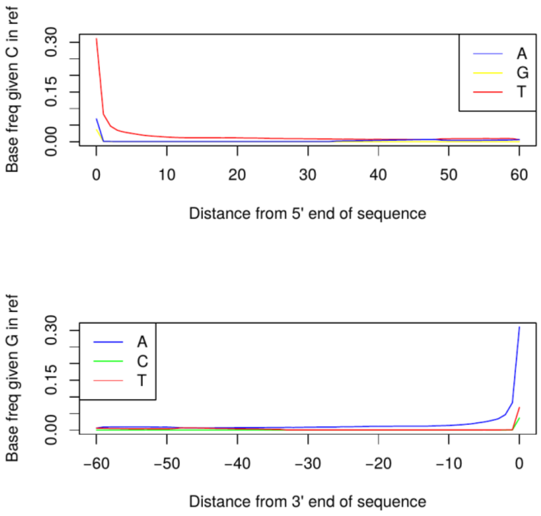

oll010

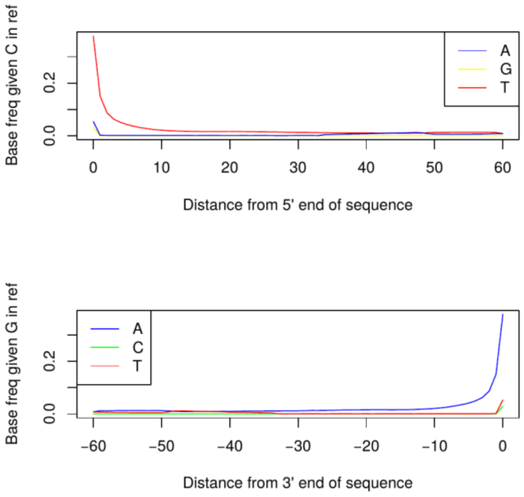

poz44

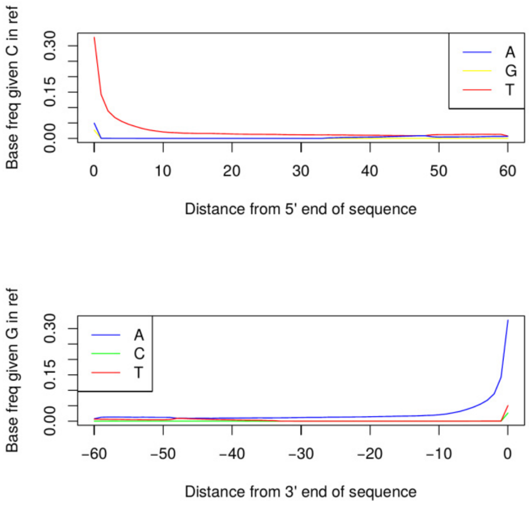

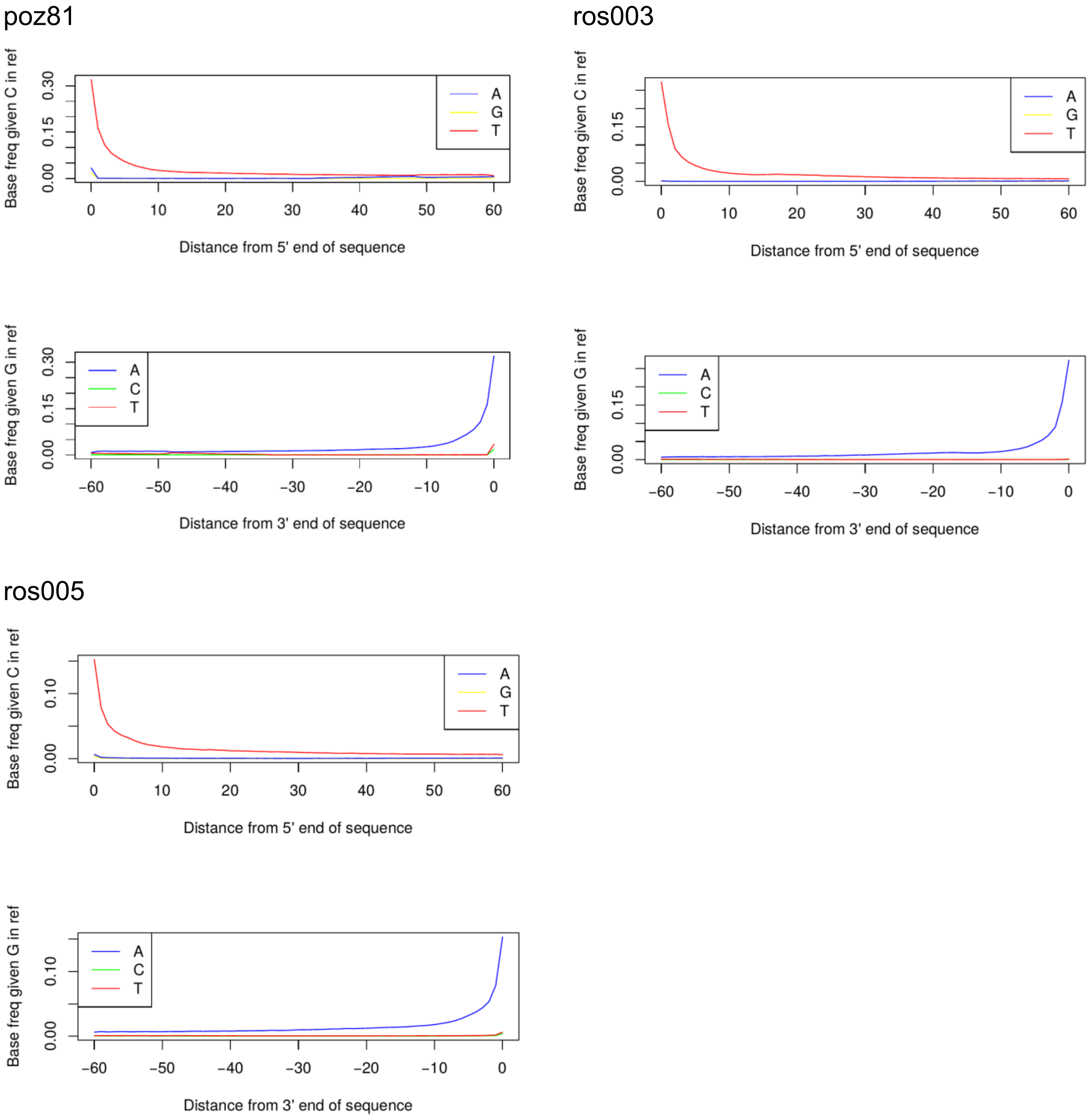

Figure S5. Deamination damage patterns (101) of the sequence reads from the eleven individuals in this study showed expected fragmentation and damage patterns as expected for authentic ancient DNA.

Contamination estimates

Mitochondrial contamination was estimated using a method first presented in Green et al. (102) (Table 1). In brief, we used private or nearly private consensus alleles in the individual and counted all alternative alleles mapping to those sites. We excluded sites with consensus allele of either C or G and observed transition alleles in order to exclude the effect of post-mortem deamination. Consensus and alternative alleles were summed up across all informative sites and divided by the total number of alleles in order to obtain a point estimate for contamination. Additionally, we used ANGSD's method 1 to obtain an X chromosome based estimate of nuclear contamination in male individuals (Table S3) (103,104).

Table S3. Contamination estimates for the eleven individuals based on mtDNA (102) and the X chromosome in males (103,104).

	mtDNA based estimation						*X based estimation*		
Sample	Point estimate	Informative sites	Consenus alleles	Total alleles	Lower C.I	Higher C.I	Cont estimated [%]	SE	# SNPs
**kar1**	0.794	3	5870	5917	0.568	1.021	n/a	n/a	n/a
**poz44**	0.287	2	348	349	0.000	0.847	n/a	n/a	n/a
**poz81**	1.323	6	746	756	0.508	2.137	1.155	0.115	34637
**ber1**	0.186	6	6452	6464	0.081	0.291	0.803	0.059	39714
**ber2**	0.256	8	2337	2343	0.051	0.461	n/a	n/a	n/a
**oll007**	0.444	2	224	225	0.000	1.314	n/a	n/a	n/a
**oll009**	1.975	5	1390	1418	1.250	2.699	0.317	0.099	20863
**oll010**	1.832	7	536	546	0.707	2.956	n/a	n/a	n/a
**ros003**	0.403	7	247	247	0.000	1.192	n/a	n/a	n/a
**ros005**	0.194	9	1031	1033	0.000	0.462	0.278	0.091	19287
**ajv54**	1.238	5	1675	1696	0.712	1.765	0.580	0.107	20470

Uniparental markers

Mitochondrial DNA

Consensus sequences were generated using samtools' mpileup and vcfutils.pl (and vcf2fq) (99) together with ANGSD (104). We used a minimum base- and mapping quality score of 30 and a coverage of at least three to call the consensus sequences. HaploFind (https://haplofind.unibo.it) (105) and PhyloTree mtDNA Build 17, 18 Feb 2016 (http://www.phylotree.org) (106) were used to call the haplogroups. The mutations are reported against the Reconstructed Sapiens Reference Sequence (107). The called haplogroups, mutations supporting the called haplogroup and private variants are reported in table S4.

The individuals in this study displayed a varied set of mitochondrial haplotypes (table S4). The two Scandinavian individuals from a typical BAC burial (ber1 and ber2) belonged to lineages within U4 and N1a while the individual dated to the BAC time-period, but buried in a megalithic tomb (oll007), belonged within hg H. Two of the continental European CWC individuals (poz81 and kar1) also belonged within sub-lineages of U4 and H, while the third CWC individual (poz44) belonged within U3. The two individuals from a Scandinavian FBC context belonged to lineages within J1 and K1 (ros003 and ros005) while the two Scandinavian Late Neolithic/Early Bronze Age individuals belonged within H and X. The individual from a PWC context (ajv54) belonged within U5.

Table S4. The called mitochondrial haplogroups for the eleven individuals in this study, including mtDNA coverage, quality scores (https://haplofind.unibo.it) (105), mutations supporting the called haplogroup and private mutations.

Sample	MT haplo-group	MT coverage	Score	Mutations supporting the called haplogroup	Private mutations
ber1	U4c1a	1344.2	1	146T 152T 247G 499A 769G 825T 1018G 1811G 2758G 2885T 3594C 3654T 4104A 4312C 4646C 4811G 5999C 6047G 6146G 7146A 7256C 7521G 8468C 8655C 8701A 9070G 9540T 10398A 10664C 10688G 10810T 10873T 10907C 10915T 11009C 11332T 11467G 11914G 12308G 12372A 12705C 13105A 13276A 13506C 13650C 14620T 14866T 15693C 16129G 16179T 16187C 16189T 16223C 16230A 16278C 16311T 16356C	12373G 16569A 4769A 8860A
ber2	N1a1a1a1	442.7	1	146T 195T 199C 204C 247G 669C 769G 825T 1018G 1719A 2758G 2885T 3336C 3594C 4104A 4312C 5315G 7146A 7256C 7521G 8164T 8468C 8655C 8701A 9300A 9540T 10238C 10664C 10688G 10810T 10873T 10915T 11914G 12501A 13105A 13276A 13506C 13650C 13780G 15043A 16129G 16147A 16172C 16187C 16189T 16230A 16248T 16278C 16311T 16320T 16355T	4769A
kar1	H1f1a	2481.1	1	73A 146T 152T 195T 247G 769G 825T 1018G 2706A 2758G 2885T 3010A 3594C 4104A 4312C 4452C 7028C 7146A 7256C 7309C 7521G 8468C 8655C 8701A 9066G 9540T 10398A 10664C 10688G 10810T 10873T 10915T 11719G 11914G 12705C 13105A 13276A 13506C 13650C 14766C 16093C 16129G 16187C 16223C 16230A 16278C 16311T	4769A 8860A
poz44	U3a'c	252.9	1	146T 150T 152T 195T 247G 769G 825T 1018G 1811G 2294G 2758G 2885T 3594C 4104A 4312C 4703C 7146A 7256C 7521G 8468C 8655C 8701A 9266A 9540T 10398A 10664C 10688G 10810T 10873T 10915T 11467G 11914G 12308G 12372A 12705C 13105A 13276A 13506C 13650C 14139G 15454C 16129G 16187C 16189T 16223C 16230A 16278C 16311T 16343G	310C 8860A 12843C 13327G 16243C 16249C 16519T 16569A
poz81	U4b1b2	172.4	1	146T 247G 499A 769G 825T 1018G 1811G 2758G 2885T 3594C 4104A 4312C 7146A 7256C 7521G 8468C 8655C 8701A 9540T 10398A 10664C 10688G 10810T 10873T 10915T 11332T 11339C 11467G 11788T 11914G 12308G 12372A 12705C 13105A 13276A 13506C 13528G 13650C 14620T 15326A 15514C 15693C 16129G 16187C 16189T 16223C 16230A 16278C 16311T 16356C	310C 4769A 4770A
ros003	K1b1a1	29.6	1	146T 195T 247G 769G 825T 1018G 1811G 2758G 2885T 3480G 3594C 4312C 7256C 8468C 8655C 8701A 9055A 9540T 9698C 9962A 10289G 10550G 10664C 10688G 10810T 10873T 10915T 11299C 11467G 11914G 11923G 12308G 12372A 12705C 13105A 13276A 13506C 13650C 13967T 14167T 14798C 15257A 15946T 16093C 16129G 16187C 16189T 16223C 16224C 16230A 16278C 16319A 16463G	310C 4252T 10310A
ros005	J1c5	105.6	1	146T 152T 185A 195T 228A 247G 295T 462T 489C 769G 825T 1018G 2758G 2885T 3010A 3594C 4104A 4216C 4312C 5198G 7146A 7256C 7521G 8468C 8655C 8701A 9540T 10664C 10688G 10810T 10873T 10915T 11251G 11914G 12612G 12705C 13105A 13276A 13506C 13650C 13708A 14798C 15452A 16069T 16126C 16129G 16187C 16189T 16223C 16230A 16278C 16311T	3666A 16519T

oll009	H6a1b3	324.7	1	73A 146T 152T 195T 204C 239C 247G 769G 825T 1018G 2706A 2758G 2885T 3594C 3915A 4104A 4312C 4727G 7028C 7146A 7256C 7521G 8468C 8655C 8701A 9380A 9540T 10398A 10589A 10664C 10688G 10810T 10873T 10915T 11719G 11914G 12705C 13105A 13276A 13506C 13650C 14766C 16129G 16187C 16189T 16193T 16219G 16223C 16230A 16278C 16311T 16362C 16482G	310C 8863T 16519T 16569A
oll010	X2b11	96.0	0.7	146T 152T 153G 189G 225A 226C 247G 769G 825T 1018G 1719A 2758G 2885T 3594C 4104A 4312C 6221C 6371T 7146A 7256C 7521G 8393T 8468C 8655C 8701A 9540T 10398A 10664C 10688G 10810T 10873T 10915T 11914G 13105A 13276A 13506C 13650C 13708A 13966G 14470C 15927A 16129G 16187C 16230A 16311T	310C 5253T
oll007	H1c	85.7	1	73A 146T 152T 195T 247G 477C 769G 825T 1018G 2706A 2758G 2885T 3010A 3594C 4104A 4312C 7028C 7146A 7256C 7521G 8468C 8655C 8701A 9540T 10398A 10664C 10688G 10810T 10873T 10915T 11719G 11914G 12705C 13105A 13276A 13506C 13650C 14766C 16129G 16187C 16189T 16223C 16230A 16278C 16311T	-
ajv54	U5b1d2	509.8	0.5	146T 150T 152T 195T 247G 769G 825T 1018G 2758G 2885T 3197C 3594C 4104A 4312C 5437T 5656G 7085C 7146A 7256C 7521G 7768G 8468C 8655C 8701A 9477A 9540T 10398A 10664C 10688G 10810T 10873T 10915T 11467G 11914G 12308G 12372A 12705C 13105A 13276A 13506C 13617C 13650C 14182C 16129G 16187C 16192T 16223C 16230A 16270T 16278C 16311T	310C 3206T 6179A 16519T

Y-chromosomal DNA

Single base substitutions from Phylotree (version 09/03/2016) (108) were called from bam files mapped to hg 19 using Samtools v.1.3 (99). Sites with a mapping- and a base quality of at least 30 were extracted. Insertions, deletions and sites with chimeric alleles were excluded as well as some sporadic derived alleles with low coverage which were contradicted by upstream ancestral alleles. The remaining derived alleles were manually examined for phylogenetic sense. We report all derived alleles for the branch of the called haplogroup in a hierarchal phylogenetic order for ber1 and poz81 while only describing the data for remaining males as there were not enough data for confident haplogroup assignments (table S5).

The Y-chromosomal data showed that ber1, a male from a BAC context, belonged to R-Z283 (xM458, Z91, Z284), a sub-lineage within hg R1a (table S5). We can exclude that he belongs to the sister lineages R-Z291 and R-Z284, as well as further down within the R-Z283 sub-lineage.

Another male, poz81, from a CWC context, also belonged within a sub-lineage of R1a, namely R-M417 (xZ645) (table S5). We cannot exclude that he belonged a bit further down in this lineage as there was no data for CTS4385 and S3485, but we can exclude R-S3485. We can also exclude him belonging within the sister lineage R-Z645.

Less Y-chromosomal data was available for the remaining three males in this study (ros005, ajv54 and oll009). Therefore, we describe this data here (with marker names, position, mutation, and number of reads in brackets) without adding the information to table S5 and caution that more data is needed to confidently call haplogroups. We also examined ancestral allele states leading to R1a haplotypes (found in ber1 and poz81) among these three individuals to further strengthen an exclusion from this lineage.

The individual ros005, a male from a FBC context, likely belong to hg IJ or possibly within

the I-lineage (IJ-M429*(xM304)). We found 10 derived mutations leading towards IJ (L1155:22191266, G>C, 1 for A0'1'2'3'4; V168:17947672, G>A, 3 for A1'2'3'4; V221:7589303, G>T, 1 for A2'3'4; P97:14886273, G>T, 1 for A4=BCDEF; M213:15526751, T>C, 2 and P14:17398598, C>T, 1 for F; M522:7173143, G>A, 1 and M523:6753519, A>G, 1 for IJKLT, and finally M429:14031334, T>A, 1 and P126:21225770, C>G, 1 for IJ). We then investigated which lineages within IJ that could be excluded. As ros005 displayed ancestral alleles for J-M304 (both for M304:22749853, A>C, 1 and for P209:19179335, T>C, 2 characterizing J), we could exclude him belonging to hg J and sub-lineages therein. We could further exclude a belonging within I1-sublineages (ancestral alleles for I1-Z131:5845252, G>A, 1; I1-DF29:3626279, A>G, 1 and I1-CTS6364:16850799, A>T, 1), but not to I1-M253*(xZ131,DF29) as we had no data for that marker. Some of the sub-lineages within I2 could likely also be excluded. These were I2-L596 (L597:18887888, T>A, 1), I2-L38 (L38:15668070, A>G, 3) and I2-M223 (M223:21717307, G>A, 1). Based on ancestral allele states for ros005, we could also verify an exclusion from the R1a-lineages found in the ber1 and poz81 males (R1-M173:15026424, A>C, 3 and R1a-Z283:21976303, T>A, 2).

Individual ajv54, a male from a PWC context, likely belong to hg I2-M438 or sub-lineages therein. We found nine derived mutations leading towards I2 (L1085:2790726, T>C, 1 for A0'1'2'3'4; V168:17947672, G>A, 1 for A1'2'3'4; M42:21866840, A>T, 1 and P97:14886273, G>T, 1 for A4=BCDEF; M213:15526751, T>C, 1 for F; M578:7202703, C>T, 1 for HIJKLT; M522:7173143, G>A, 1 for IJKLT; U179:16354708, G>A, 2 for I and M438:16638804, A>G, 1 for I2). As the amount of data is quite low, and to further strengthen that ajv54 did not belong within the R1a-lineages found in ber1 and poz81 males, we list key ancestral allele states leading towards R1a (K-M526:23550924, A>C, 2; P-P295:7963031, T>G, 1; QR-M74:21889767, G>A, 2; R-M207:15581983, A>G, 2 and R1a-Z645:8245045, C>T, 2).

The individual oll9, a Scandinavian Late Neolithic male from a megalithic tomb, may belong to hg I1-M253. We found 10 mutations leading in this direction (L1085:2790726, T>C, 1 and L1155:22191266, G>C, 1 for A0'1'2'3'4; V168:17947672, G>A, 1 for A1'2'3'4; P108:15426248, C>T, 1 for A2'3'4; M42:21866840, A>T, 4 and P97:14886273, G>T, 1 for A4=BCDEF; M168:14813991, C>T, 1 for CDEF; P14:17398598, C>T, 2 for F; M522:7173143, G>A, 1 for IJKLT and finally M253:15022707, C>T, 1 for I1). We note that I1-M253 is only supported by one read involving a C to T transition. However, ancestral allele states for downstream I1-Z131 (Z131:5845252, G>A, 1) and for markers defining hg J (P209:19179335, T>C, 2), M (P399:13563083, C>T, 2 and PR2099:16328755, C>T, 1), QR (M45:21867787, G>A, 1), R1a (M420:23473201, T>A, 1 and M448:16520444, T>A, 1) and LT (P326:8467290, T>C, 1) indicate that this individual likely does not belong much further down in the phylogeny.

Table S5. Y-chromosomal support for the called haplogroups for the ber1 and poz81 males including markers, marker positions in hg19, type of mutation, observed allele, number of reads and allele state (ancestral or derived).

Sample	Marker	Hg Phylotree	RefSNP ID	Position (GRCh37)	Mutation	Obs. allele	Reads	State
ber1	M173	R	rs2032624	15026424	A>C	C	2	derived
	M306	R	rs1558843	22750583	C>A	A	3	derived
	M420	R	rs17250535	23473201	T>A	A	1	derived
	M448	R	rs17222202	16520444	T>A	A	1	derived
	M198	R	rs2020857	15030752	C>T	T	2	derived
	M417	R	rs17316771	8533735	G>A	A	2	derived
	Z647	R	rs112284571	7683058	G>A	A	3	derived
	Z645	R	rs111731595	8245045	C>T	T	1	derived
	Z283	R	rs112309702	21976303	T>A	A	3	derived
	M458	R	rs375323198	24366464	A>G	A	3	ancestral
	L260	R	-	18248830	G>T	G	2	ancestral
	Z91	R	rs111908460	16474793	G>A	G	5	ancestral
	Z284	R	-	8717196	C>G	C	5	ancestral
poz81	M173	R	rs2032624	15026424	A>C	C	2	derived
	M306	R	rs1558843	22750583	C>A	A	1	derived
	M420	R	rs17250535	23473201	T>A	A	2	derived
	M198	R	rs2020857	15030752	C>T	T	2	derived
	M417	R	rs17316771	8533735	G>A	A	1	derived
	S2863	R	-	16614118	C>G	C	3	ancestral
	Z645	R	rs111731595	8245045	C>T	C	2	ancestral
	Z647	R	rs112284571	7683058	G>A	G	3	ancestral

Phenotypic traits

We investigated variants associated with physical or metabolic phenotypes by investigating lactase persistence (109) and pigmentation (110) in our samples. For lactase persistence we report read counts for rs4988235 which were assessed using samtools mpileup with minimum mapping and base qualities of 30. To predict pigmentation phenotypes, we used the HIrisplex-S system which predicts eye, hair and skin color based on 41 SNPs (https://hirisplex.erasmusmc.nl/) (110). We excluded the insertion polymorphism

rs312262906 from the prediction. If read(s) supporting only one allele were found, the individual was assumed homozygous, if both were found heterozygous or the site was called as missing data otherwise.

Table S6. Observed alleles for -13910 LCT (rs4988235) (109) and probability of predicted eye, skin and hair pigmentation from the HIrisplex-S system (https://hirisplex.erasmusmc.nl/) (110).

	**ajv54**	**ber1**	**ber2**	**kar1**	**oll007**	**oll009**	**oll010**	**poz44**	**poz81**	**ros005**	**ros003**
**Observed LCT alleles**	NA	C (6)	C (1)	T (3)	NA	C (2)	NA	NA	C (4)	C (1)	C (1)
**PBlueEye**	NA	0.01890	NA	0.25078	0.10199	NA	NA	NA	0.00192	0.90625	NA
**PIntermediate Eye**	NA	0.04039	NA	0.16305	0.13449	NA	NA	NA	0.04004	0.06073	NA
**PBrownEye**	NA	0.94071	NA	0.58617	0.76352	NA	NA	NA	0.95804	0.03302	NA
**PBlondHair**	NA	0.01740	NA	0.37060	0.35361	NA	NA	NA	0.02362	NA	NA
**PBrownHair**	NA	0.26225	NA	0.44855	0.50050	NA	NA	NA	0.56496	NA	NA
**PRedHair**	NA	0.00028	NA	0.13828	0.00097	NA	NA	NA	0.00154	NA	NA
**PBlackHair**	NA	0.72007	NA	0.04256	0.14492	NA	NA	NA	0.40988	NA	NA
**PLightHair**	NA	0.09604	0.59199	0.93021	0.68909	NA	NA	NA	0.02510	0.91355	NA
**PDarkHair**	NA	0.90396	0.40801	0.06979	0.31091	NA	NA	NA	0.97490	0.08645	NA
**PVeryPaleSkin**	0.11880	0.24211	0.13852	0.10972	0.04923	0.02848	NA	NA	0.00711	0.21828	NA
**PPaleSkin**	0.68342	0.17187	0.29553	0.28068	0.10585	0.14287	NA	NA	0.00798	0.06854	NA
**PIntermediate Skin**	0.19714	0.58542	0.50079	0.60960	0.80395	0.78385	NA	NA	0.69662	0.52722	NA
**PDarkSkin**	0.00061	0.00000	0.05794	0.00000	0.02917	0.04177	NA	NA	0.20951	0.17152	NA
**PDarktoBlack Skin**	0.00003	0.00059	0.00722	0.00000	0.01180	0.00303	NA	NA	0.07878	0.01444	NA

Comparative genome data

We downloaded data for published ancient individuals and processed them through the same pipeline as outlined above to reduce technical artefacts. We included individuals with at least 0.1x coverage according to the original publication and who lived at the time of the CWC or before to include potential ancestral and contemporary groups for the newly sequenced BAC and CWC individuals. All individuals are listed in table S7 (38–41,48,49,78,80,81,96,111–122). For each ancient individual and each investigated biallelic SNP site, we randomly sampled a read with mapping quality of at least 30. We used samtools mpielup with the option -B to turn off base quality rescaling for this step. If this read carried one of the two known alleles with a base quality of at least 30, we used it as pseudo-haploid representation of the individual. For non-UDG treated individuals, we excluded transition sites to exclude the effect of post-mortem deamination.

We compared the ancient individuals to a set of modern population from the Human Origins dataset (114) and the public data of the Simons Genome Diversity Panel (123).

Table S7. List of comparative aDNA data (n=272) and the individuals from this study (n=11) including sample name, cultural contexts (abbreviations are AFA=Afanasievo, ALP=Alföld Linear Pottery, AND=Andronovo, BA=Bronze Age, BAA=Baalberge, BAC=Battle Axe, BAD=Baden, BBC=Bell Beaker, BER=Bernburg, BUK=Bükk, CAR=Cardial Ware, CCC=Combed Ware, CHA=Chalcolithic, CWC=Corded Ware, EBA=Early Bronze Age, EMN=Early Middle Neolithic, ENE=Eneolithic, EPI=Epicardial, FBC=Funnel Beaker, HG=Hunter-gatherer, KAR=Karasuk, KOR=Körös, KYJ=Kyjatice, LBA=Late Bronze Age, LBK=Linear Band, LEN=Lengyel, LN=Late Neolithic, MAK=Makó, MAR=Maros, MEG=Megalithic burials, MEZ=Mezhovskaya, MN=Middle Neolithic, NEO=Neolithic Turkey, OKU=Okunevo, POL=Poltavka, POT=Potapovka, PWC=Pitted Ware, REM=Remedello, SIN=Sintashta, SRU=Srubnaya, STA=Starčevo, TIS=Tiszadob-Bükk, UNE=Únětice, URN=Urnfield, VAT=Vatya, YAM=Yamnaya), International Organization for Standardization (ISO) country codes and references.

Individual	Context	Cou ntry	Reference
RISE508	AFA	RU	(48)
RISE509	AFA	RU	(48)
RISE510	AFA	RU	(48)
RISE511	AFA	RU	(48)
I1498	ALP	HU	(113,119)
I1500	ALP	HU	(113,119)
I1506	ALP	HU	(113,119)
RISE500	AND	RU	(48)
RISE503	AND	RU	(48)
RISE505	AND	RU	(48)
RISE512*	ANDo	RU	(48)
RISE397	BA	AM	(48)
RISE407	BA	AM	(48)
RISE423	BA	AM	(48)
I0047	BA	DE	(49,119)
RISE47	BA	DK	(48)
ATP9	BA	ES	(96)
Rathlin1	BA	IE	(117)
Rathlin2	BA	IE	(117)
Rathlin3	BA	IE	(117)
RISE431	BA	PL	(48)
I0559	BAA	DE	(49,119)
I0560	BAA	DE	(49,119)
ber1	BAC	SE	*This study*
ber2	BAC	SE	*This study*
RISE94	BAC	SE	(48)
I1497	BAD	HU	(113,119)
RISE569	BBC	CZ	(48)
I0059	BBC	DE	(49,119)
I0060	BBC	DE	(49,119)
I0108	BBC	DE	(49,119)
I0111	BBC	DE	(49,119)
I0113	BBC	DE	(49,119)
I0805	BBC	DE	(119)
I0806	BBC	DE	(49,119)
I1546	BBC	DE	(119)
I1549	BBC	DE	(119)
RISE559	BBC	DE	(48)
RISE563	BBC	DE	(48)
I0112	BBCo	DE	(49,119)
I0172	BER	DE	(49,119)
I1499	BUK	HU	(119)
CB13	CAR	ES	(120)
Kudrukula3	CCC	EE	(40)
Tamula1	CCC	EE	(39)
Tamula3	CCC	EE	(39)
ATP12-1420	CHA	ES	(96)
ATP16	CHA	ES	(96)
ATP2	CHA	ES	(96)
I1271	CHA	ES	(119)
I1272	CHA	ES	(119)
I1276	CHA	ES	(119)
I1277	CHA	ES	(119)
I1280	CHA	ES	(119)
I1281	CHA	ES	(119)
I1284	CHA	ES	(119)
I1300	CHA	ES	(119)
I1303	CHA	ES	(119)
I1314	CHA	ES	(119)
I0049	CWC	DE	(49,119)
I0103	CWC	DE	(49,119)
I0104	CWC	DE	(49,119)
I0106	CWC	DE	(49,119)
I1532	CWC	DE	(119)
I1534	CWC	DE	(119)
I1536	CWC	DE	(119)
I1538	CWC	DE	(119)
I1539	CWC	DE	(119)

I1540	CWC	DE	(119)
I1544	CWC	DE	(119)
RISE446	CWC	DE	(48)
Ardu1r	CWC	EE	(40)
Ardu2	CWC	EE	(40)
kar1	CWC	EE	*This study*
Kunila1	CWC	EE	(40)
Kunila2	CWC	EE	(40)
RISE00	CWC	EE	(48)
ZVEJ28	CWC	LV	(38)
Gyvakarai1	CWC	LT	(39)
Plinkaigalis2 41	CWC	LT	(39)
Plinkaigalis2 42	CWC	LT	(39)
N44	CWC	PL	(41)
N45	CWC	PL	(41)
N47	CWC	PL	(41)
N49	CWC	PL	(41)
poz44	CWC	PL	*This study*
poz81	CWC	PL	*This study*
RISE555	EBA	RU	(48)
Kivisaare3	EMN	EE	(39)
Veibri4	EMN	EE	(39)
Donkalnis6	EMN	LT	(39)
Donkalnis7	EMN	LT	(39)
Kretuonas1	EMN	LT	(39)
Kretuonas2	EMN	LT	(39)
Kretuonas4	EMN	LT	(39)
Kretuonas5	EMN	LT	(39)
Spiginas1	EMN	LT	(39)
I0122	ENE	RU	(119)
I0409	EPI	ES	(49,119)
I0410	EPI	ES	(49,119)
I0412	EPI	ES	(49,119)
I0413	EPI	ES	(49,119)

N18	FBC	PL	(41)
N19	FBC	PL	(41)
N20	FBC	PL	(41)
gok2	FBC	SE	(81)
ros003	FBC	SE	*This study*
ros005	FBC	SE	*This study*
Saxtorp5164	FBC	SE	(39)
Bichon	HG	CH	(118)
I1875	HG	CR	(122)
Falkenstein	HG	DE	(122)
I0585	HG	ES	(119,120)
LaBrana1	HG	ES	(120,124)
Iboussieres2 5-1	HG	FR	(122)
Kotias	HG	GE	(118)
Satsurblia	HG	GE	(118)
Spiginas4	HG	LT	(39)
Loschbour	HG	LU	(125)
ZVEJ25	HG	LV	(38)
ZVEJ27	HG	LV	(38)
ZVEJ32	HG	LV	(38)
H22	HG	NO	(78)
H26	HG	NO	(78)
I0061	HG	RU	(49,119)
I0124	HG	RU	(49,119)
I0211	HG	RU	(119)
UzOO77	HG	RU	(39)
I0011	HG	SE	(49,119)
I0012	HG	SE	(49,119)
I0013	HG	SE	(49,119)
I0014	HG	SE	(49,119)
I0015	HG	SE	(49,119)
I0016	HG	SE	(49)
I0017	HG	SE	(49,119)
sbj001	HG	SE	(78)

sf11	HG	SE	(78,80)
sf12	HG	SE	(78)
sf9	HG	SE	(78)
stg001	HG	SE	(78)
StPet12	HG	UA	(38)
Iceman	Iceman	IT	(111)
RISE493	KAR	RU	(48)
RISE494	KAR	RU	(48)
RISE495	KAR	RU	(48)
RISE496	KAR	RU	(48)
RISE497	KAR	RU	(48)
RISE499	KAR	RU	(48)
RISE502	KAR	RU	(48)
I1507	KO1	HU	(113,119)
I1508	KOR	HU	(113,119)
Kostenki	Kostenki	RU	(115)
I1504	KYJ	HU	(113)
RISE554	LBA	RU	(48)
I0022	LBK	DE	(49,119)
I0025	LBK	DE	(49,119)
I0026	LBK	DE	(49,119)
I0046	LBK	DE	(49,119)
I0048	LBK	DE	(119)
I0054	LBK	DE	(49,119)
I0057	LBK	DE	(49,119)
I0100	LBK	DE	(49,119)
I0659	LBK	DE	(49,119)
I0821	LBK	DE	(49,119)
I1550	LBK	DE	(119)
Stuttgart	LBK	DE	(125)
I1496	LBK	HU	(113,119)
I1495	LEN	HU	(113,119)
I0058	LN	DE	(49)
I0118	LN	DE	(49,119)
I0171	LN	DE	(49,119)

I0550	LN	DE	(49,119)
RISE71	LN	DK	(48)
oll009	LN	SE	*This study*
oll010	LN	SE	*This study*
Olsund	LN	SE	(39)
RISE97	LN	SE	(48)
RISE98	LN	SE	(48)
I1502	MAK	HU	(113,119)
Mal´ta	Malta	RU	(116)
RISE373	MAR	HU	(48)
RISE374	MAR	HU	(48)
I0405	MEG	ES	(49,119)
I0406	MEG	ES	(49,119)
I0407	MEG	ES	(49,119)
I0408	MEG	ES	(49,119)
Ballynahatty	MEG	IE	(117)
RISE523	MEZ	RU	(48)
RISE525	MEZ	RU	(48)
RISE61	MN	DK	(48)
ZVEJ26	MN	LV	(38)
ZVEJ31	MN	LV	(38)
oll007	MN	SE	*This study*
I0707	NEO	TR	(119)
I0708	NEO	TR	(119)
I0709	NEO	TR	(119)
I0723	NEO	TR	(119)
I0726	NEO	TR	(119)
I0736	NEO	TR	(119)
I0744	NEO	TR	(119)
I0745	NEO	TR	(119)
I0746	NEO	TR	(119)
I1096	NEO	TR	(119)
I1097	NEO	TR	(119)
I1098	NEO	TR	(119)
I1099	NEO	TR	(119)

I1100	NEO	TR	(119)
I1101	NEO	TR	(119)
I1102	NEO	TR	(119)
I1103	NEO	TR	(119)
I1579	NEO	TR	(119)
I1580	NEO	TR	(119)
I1581	NEO	TR	(119)
I1583	NEO	TR	(119)
I1585	NEO	TR	(119)
Kum6	NEO	TR	(126)
StPet2	NEO	UA	(38)
RISE515	OKU	RU	(48)
RISE516	OKU	RU	(48)
I0126	POL	RU	(119)
I0371	POL	RU	(119)
I0374	POL	RU	(119)
I0433	POL	RU	(119)
I0440	POL	RU	(119)
I0432*	POLo	RU	(119)
I0418	POT	RU	(119)
I0419	POT	RU	(119)
ajv54	PWC	SE	*This study*
ajv58	PWC	SE	(78,80)
ajv70	PWC	SE	(78,80)
RISE486	REM	IT	(48)
RISE487	REM	IT	(48)
RISE489	REM	IT	(48)
RISE386	SIN	RU	(48)
RISE392	SIN	RU	(48)
RISE394	SIN	RU	(48)
RISE395	SIN	RU	(48)
I0232	SRU	RU	(119)
I0234	SRU	RU	(119)
I0235	SRU	RU	(119)

I0354	SRU	RU	(119)
I0358	SRU	RU	(119)
I0359	SRU	RU	(119)
I0361	SRU	RU	(119)
I0422	SRU	RU	(119)
I0423	SRU	RU	(119)
I0424	SRU	RU	(119)
I0430	SRU	RU	(119)
I0174	STA	HU	(49,119)
I1505	TIS	HU	(113,119)
RISE577	UNE	CZ	(48)
RISE586	UNE	CZ	(48)
I0115	UNE	DE	(49,119)
I0116	UNE	DE	(49,119)
I0117	UNE	DE	(49,119)
I0164	UNE	DE	(49,119)
I0803	UNE	DE	(49,119)
RISE109	UNE	PL	(48)
RISE150	UNE	PL	(48)
RISE154	UNE	PL	(48)
I0099	URN	DE	(49,119)
Ust	UstIshim	RU	(112)
RISE247	VAT	HU	(48)
RISE479	VAT	HU	(48)
RISE480	VAT	HU	(48)
RISE484	VAT	HU	(48)
I0231	YAM	RU	(49,119)
I0357	YAM	RU	(49,119)
I0370	YAM	RU	(49,119)
I0429	YAM	RU	(49,119)
I0438	YAM	RU	(49,119)
I0439	YAM	RU	(49,119)
I0443	YAM	RU	(49,119)

I0444	YAM	RU	(49,119)
RISE240	YAM	RU	(48)
RISE546	YAM	RU	(48)
RISE547	YAM	RU	(48)
RISE548	YAM	RU	(48)
RISE550	YAM	RU	(48)
RISE552	YAM	RU	(48)

Population genetic analyses

Principal component analyses

We used smartpca to conduct a principal component analysis (PCA) of modern Western Eurasians from the Human Origins panel. The ancient individuals were projected onto the PC space employing lsqproject: YES and shrinkmode: YES.

Unsupervised admixture

In order to perform model-based clustering, we used the software ADMIXTURE (127). We ran the software after LD pruning the full Human Origins data set together with all ancient individuals using plink and the parameters --indep-pairwise 200 25 0.4. ADMIXTURE was run for K ranging from 2 to 15 with 20 iterations per value of K. Joint modes across the independent runs were found using pong in greedy mode and displayed using GNU R.

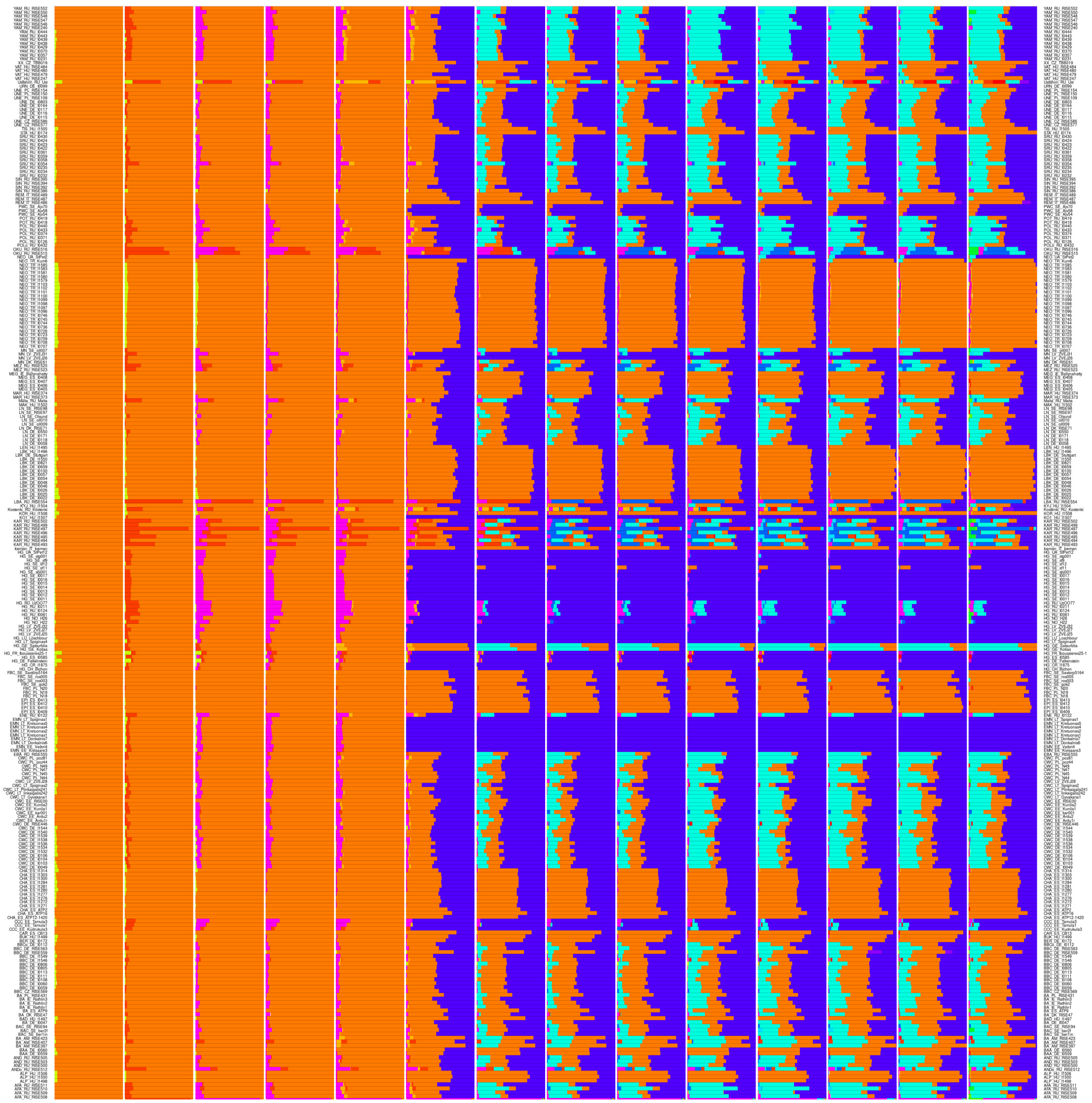

Figure S6. Genomic composition of eleven ancient individuals from this study and 272 previously published ancient individuals (see table S7) using unsupervised Admixture (127).

f4 statistic and Admixture graphs

In order to investigate source populations and admixture proportions we used ADMIXTOOLS' qpWave and qpAdm (versions 400 and 650) (49,128). We employed UstIshim_RU_Ust, Villabruna, Mixe, Natufian, Iran_Neolithic, Mbuti, CHG, Malta_RU_Malta, Kostenki_RU_Kostenki as outgroups and used all autosomal sites included in the 1240k SNP capture data. Additional ancient outgroup individuals were obtained from Lazaridis et al. (129), data for modern populations was obtained from the SGDP panel (123). Additionally, we used qpGraph (version 6100) to reconstruct admixture graphs. These were based on transversions

only and by using the Mbuti from the SGDP data as an outgroup. We only included CWC models older than 2600 cal BCE in the models and excluded models with less than 15000 SNPs due to low power for rejection. Tested models with worst |Z|<3 were considered consistent with the data (Figures 3, S7 and S8).

popstats (130) was used to calculate f4 statistics across all autosomal sites included in the 1240k SNP capture data (119) and the chimp reference genome as an outgroup. The comparative data used in this study consists of published data from a number of different studies (Table S7). Two main types of data are prevalent in the aDNA research field: whole genome shotgun sequencing and genome-wide SNP capture. The different wet lab procedures used for producing the data introduce the potential of methodological biases due to different mapping properties. aDNA data generated with both different strategies can display affinities due to a shared experimental methodology and not to a recent shared demographic history. This can particularly impact analyses involving direct pairwise comparisons between individuals such as f statistics and related measures [see e.g. (78,131)]. Therefore, out f4 analysis could potentially be affected by such biases even though the strong genetic differences between the reference groups YAM and LBK should be stronger than such technical biases. We still wanted to test if our results still hold when accounting for potential spurious allele sharing due to similar wet lab procedures used to produce some individuals. We repeat the analysis shown in Figure 2 but only use SNP capture data (Figure S9) or shotgun data (Figure S10) for the reference populations YAM and LBK. Reducing the sample sizes for the reference populations led to higher uncertainties in general as indicated by the longer error bars. Nevertheless, we observe that all regression lines show a positive slope so we would always have arrived at the conclusion that YAM ancestry reduces over time regardless of what type of data is used for the analysis. Our conclusions do not seem to be affected by potential technical artifacts related to the type of data used in the analyses.

Table S8. The BAC and CWC samples used for Figures 2, S9 and S10. The dates are presented as reported in the original publications for each sample [see table S7 for references and (132) for RISE00], either as calibrated radiocarbon dates (cal BCE), with 95% or 68% CI, or estimated based on archaeological contexts (BCE). It was not possible to re-calibrate all radiocarbon dated samples with 95% CI, as some of the original publications had not reported conventional radiocarbon ages BP. For the conclusions in Figure 2, we use the mid-points of the reported time intervals as they should not be sensitive to the differences in CI between the samples. Data type indicates if the data was generated using shotgun sequencing (SG) or capture (CAP).

Sample ID	Site	Contry	Date lower	Date upper	CI		Data type
BAC_SE_ber1	Linköping	Sweden	2620	2470	95%	cal BCE	SG
BAC_SE_ber2	Linköping	Sweden	2640	2480	95%	cal BCE	SG
BAC_SE_RISE94	Viby	Sweden	2621	2472	95%	cal BCE	SG
CWC_DE_I0049	Esperstedt	Germany	2464	2210	68%	cal BCE	CAP
CWC_DE_I0103	Esperstedt	Germany	2578	2468	68%	cal BCE	CAP
CWC_DE_I0104	Esperstedt	Germany	2559	2296	68%	cal BCE	CAP
CWC_DE_I0106	Esperstedt	Germany	2464	2210	68%	cal BCE	CAP
CWC_DE_I1532	Esperstedt	Germany	2500	2050		BCE	CAP
CWC_DE_I1534	Esperstedt	Germany	2500	2050		BCE	CAP
CWC_DE_I1536	Esperstedt	Germany	2500	2050		BCE	CAP
CWC_DE_I1538	Esperstedt	Germany	2500	2050		BCE	CAP
CWC_DE_I1539	Esperstedt	Germany	2625	2291	95%	cal BCE	CAP
CWC_DE_I1540	Esperstedt	Germany	2500	2050		BCE	CAP
CWC_DE_I1544	Esperstedt	Germany	2500	2050		BCE	CAP
CWC_DE_RISE446	Bergrheirfeld	Germany	2829	2465	95%	cal BCE	SG
CWC_EE_Ardu2	Ardu	Estonia	2921	2555	95%	cal BCE	SG
CWC_EE_kar1	Karlova	Estonia	2460	2130	9%	cal BCE	SG
CWC_EE_Kunila2	Kunila	Estonia	2626	2390	95%	cal BCE	SG
CWC_EE_RISE00	Sope	Estonia	2575	2349	95%	cal BCE	SG
CWC_LT_Gyvakarai1	Gyvakarai	Lithuania	2620	2470	95%	cal BCE	CAP
CWC_LT_Plinkaigalis242	Plinkaigalis	Lithuania	3260	2630	95%	cal BCE	CAP
CWC_LT_Plinkaigalis241	Plinkaigalis	Lithuania	2860	2410	95%	cal BCE	CAP
CWC_LV_ZVEJ28	Zvejnieki	Latvia	3089	2676	95%	cal BCE	SG
CWC_PL_N44	Pikutkowo	Poland	2570	2340	95%	cal BCE	SG
CWC_PL_N45	Pikutkowo	Poland	2570	2340	95%	cal BCE	SG
CWC_PL_N47	Pikutkowo	Poland	2570	2340	95%	cal BCE	SG
CWC_PL_N49	Pikutkowo	Poland	2570	2340	95%	cal BCE	SG
CWC_PL_poz44	Obłaczkowo	Poland	2865	2578	95%	cal BCE	SG
CWC_PL_poz81	Obłaczkowo	Poland	2880	2628	95%	cal BCE	SG

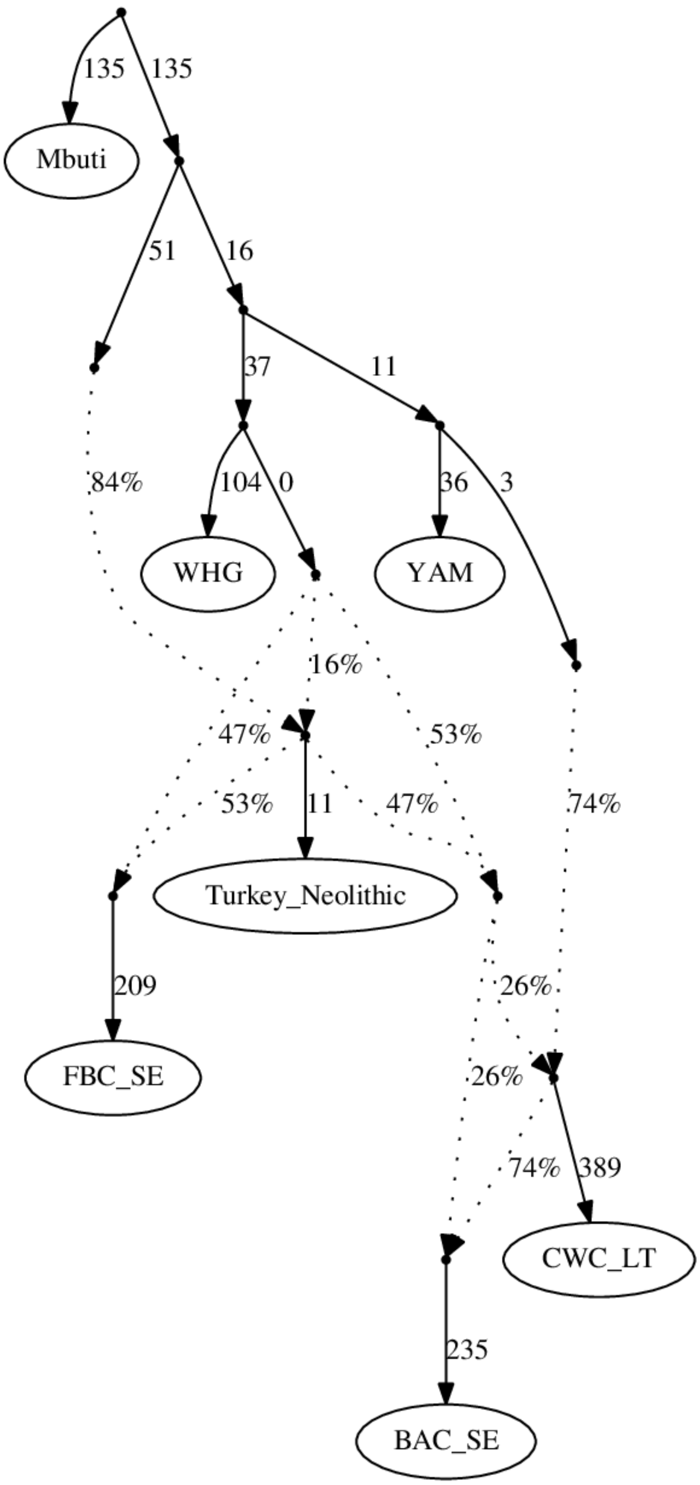

Figure S7. Admixture graph using CWC individuals from Latvia (CWC_LT) as source for BAC individuals from Sweden (BAC_SE), worst |Z|=1.7.

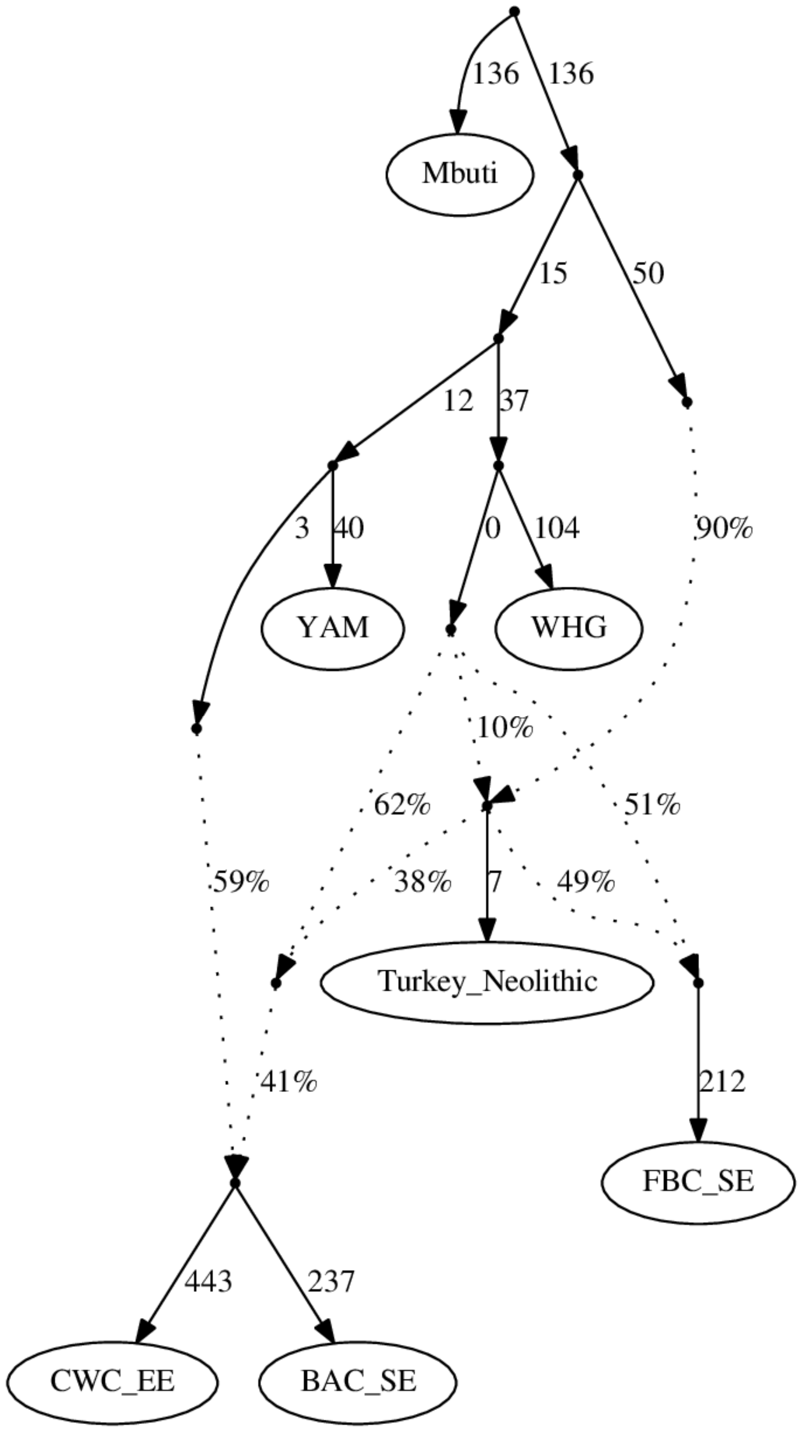

Figure S8. Admixture graph using CWC individuals from modern day Estonia (CWC_EE) as sister group to BAC individuals from Sweden (BAC_SE), worst |Z|=2.3.

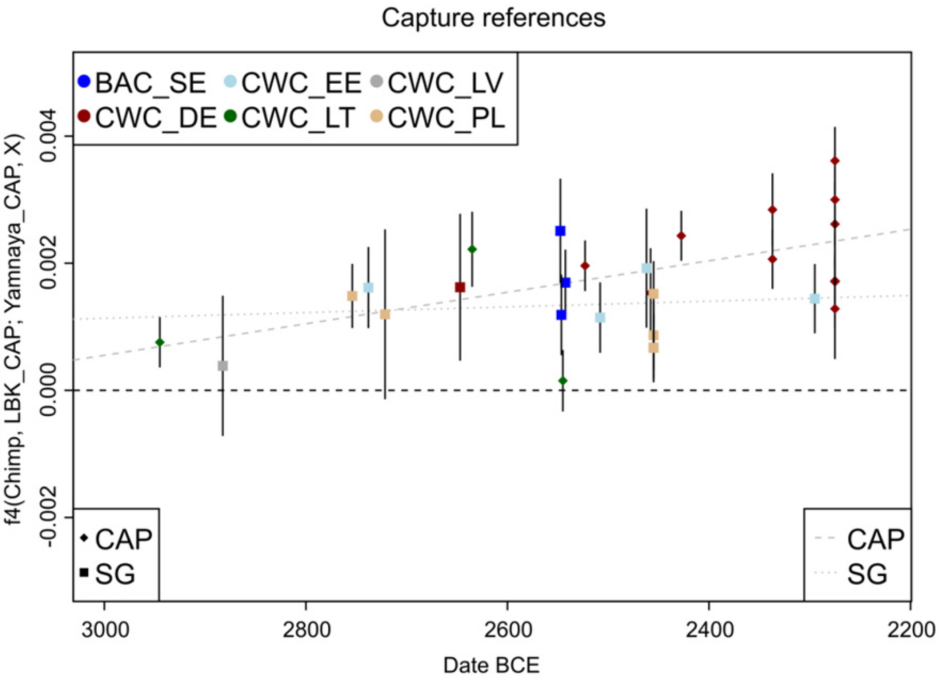

Figure S9. Similar analysis as in figure 2 but using only SNP captured data for the reference populations LBK and YAM. For each individual X, we indicated the data type in its symbol shape. Regression lines were calculated for SNP capture and shotgun individuals separately.

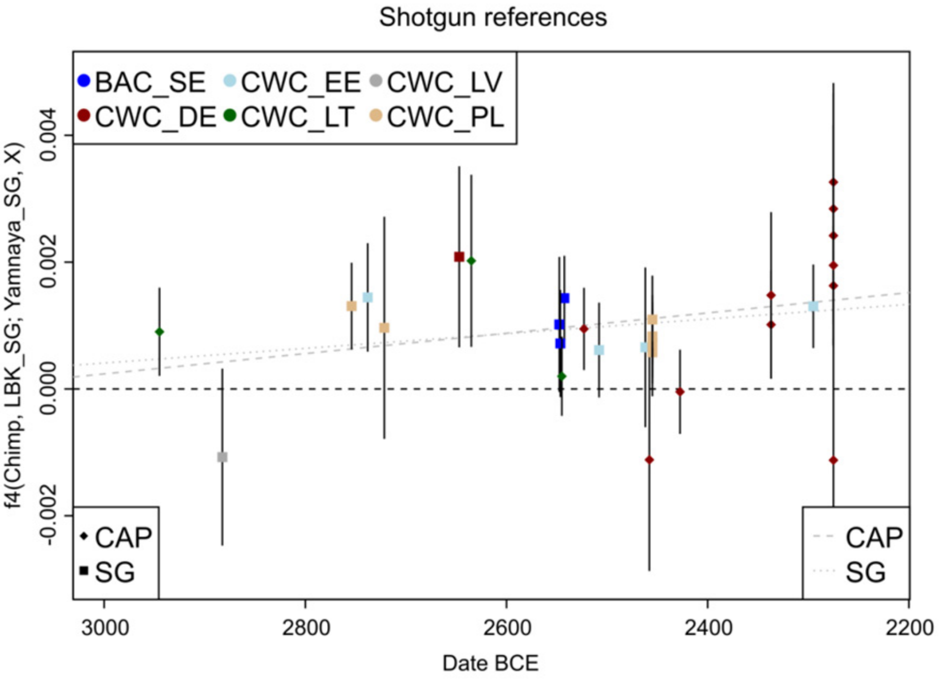

Figure S10. Similar analysis as in figure 2 but using only shotgun sequenced data for the reference populations LBK and YAM. For each individual X, we indicated the data type in its symbol shape. Regression lines were calculated for SNP capture and shotgun individuals separately.

Kinship analysis

We ran READ (133) on all shotgun-sequenced CWC and BAC individuals to investigate kinship and potentially duplicated individuals (figure S11). Only the individuals poz44 and poz81 were inferred to be second degree relatives. It is difficult to verify this degree of relationship, as we do not have genetic data from any unrelated individuals buried at Obłaczkowo, making it impossible to establish a baseline for this classification. Using all CWC individuals for this baseline comes with the risk of overestimating the expected distance between two unrelated individuals, which could overestimate their degree of relationship. Nevertheless, these two individuals show a much higher pairwise similarity than any other pair of CWC individuals (figure S11), suggesting that they might be closely related.

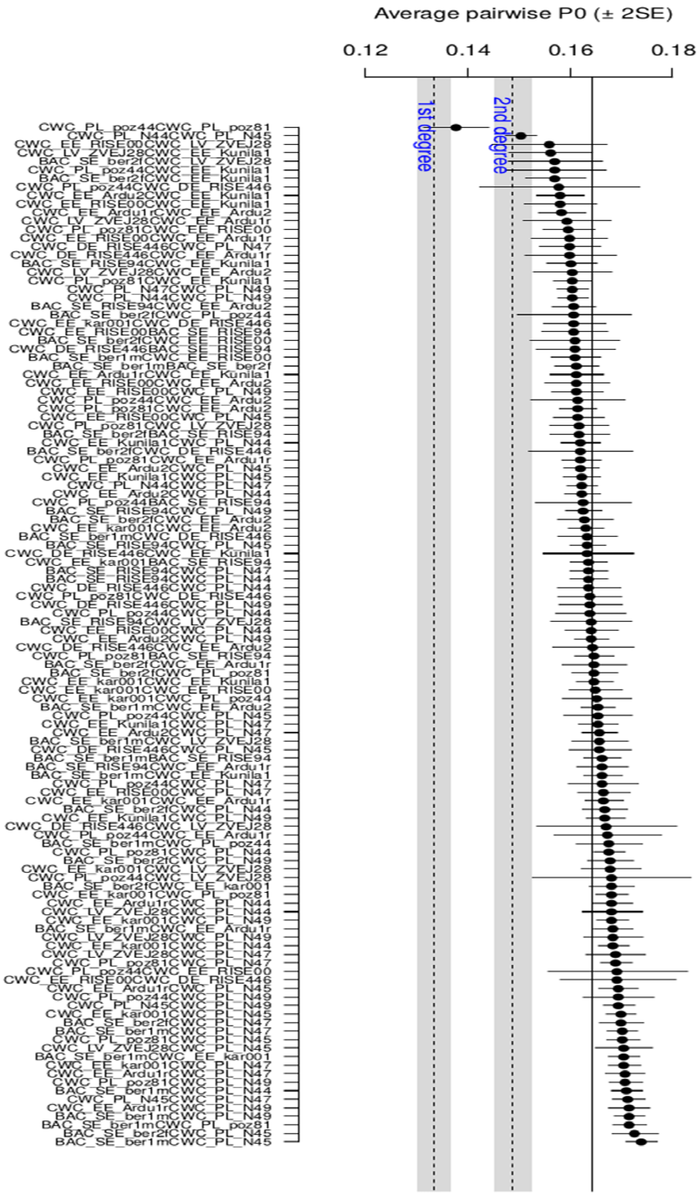

Figure S11. Kinship estimation using READ´s (133) average P0 value for all pairwise comparisons between BAC and CWC individuals.

References

1. Furholt M. Upending a 'Totality': Re-evaluating Corded Ware Variability in Late Neolithic Europe. Proc Prehist Soc. 2014 Dec;80:67–86.

2. Furholt M. Massive Migrations? The Impact of Recent aDNA Studies on our View of Third Millennium Europe. Eur J Archaeol. 2018;21(2):159–91.

3. Kristiansen K, Allentoft ME, Frei KM, Iversen R, Johannsen NN, Kroonen G, et al. Re-theorising mobility and the formation of culture and language among the Corded Ware Culture in Europe. Antiquity. 2017;91(356):334–47.

4. Edenmo R. Prestigeekonomi under yngre stenåldern: gåvoutbyten och regionala identiteter i den svenska båtyxekulturen. Uppsala: Institutionen för arkeologi och antik historia, Uppsala universitet; 2008. 298 p. (Occasional papers in archaeology 43).

5. Furholt M. Absolutchronologie und die Entstehung der Schnurkeramik. J Neolit Archaeol. 2003;[S.l.], feb. 2012. ISSN 2197-649X. Available at: <http://www.jna.uni-kiel.de/index.php/jna/article/view/7>.

6. Furholt M. Die absolutchronologische Datierung der Schnurkeramik in Mitteleuropa und Südskandinavien. Bonn: Zugl.: Bamberg, Univ., Magisterarbeit, 2002; 2003. 282 p. (Universitätsforschungen zur prähistorischen Archäologie).

7. Holmqvist E, Larsson ÅM, Kriiska A, Palonen V, Pesonen P, Mizohata K, et al. Tracing grog and pots to reveal Neolithic Corded Ware Culture contacts in the Baltic Sea region (SEM-EDS, PIXE). J Archaeol Sci. 2018 Mar;91:77–91.

8. Kriiska A, Gerasimov DV, Nordqvist K, Lisitsyn SN, Sandel S, Kholkina MA. Stone age research in the Narva-Luga Klint bay area in 2005-2014. In: New Sites, New Methods, Proceedings of the Finnish-Russian Archaeological Symposium, Helsinki, 19-21 November, 2014. Helsinki: The Finnish Antiquarian Society; 2016. p. 101–15. (Iskos 11).

9. Malmer MP. Jungneolithische Studien. [Lund]: Lund University; 1962.

10. Badou E. Finns stridsyxekultur i Norrland? In: Stridsyxekultur i Sydskandinavien. University of Lund; 1989. p. 103–9. (Larsson L, editor. Institute of Archaeology Report Series 36).

11. Knutsson K. Making and using stone tools: the analysis of the lithic assemblages from Middle Neolithic sites with flint in Västerbotten, northern Sweden. Uppsala: Societas archaeologica Upsaliensis : Dept. of Archaeology, Uppsala University; 1988. 206 p. (Aun 11).

12. Nordqvist K, Häkälä P. Distribution of Corded Ware in the Areas North of the Gulf of Finland - an Update. Est J Archaeol Tallinn. 2014;18(1):3–29.

13. Kriiska A. Corded Ware Culture sites in North-eastern Estonia. In: Jaanits L, Lang V, editors. De Temporibus Antiquissimis Ad Honorem Lembit Jaanits. 2000. p. 59–79. (Muinasaja Teadus 8).

14. Brink K. I palissadernas tid. Om stolphål och skärvor och sociala relationer under yngre mellanneolitikum. Malmö: Malmö Museer Arkeologienheten; 2009. 427 p. (Malmöfynd).

15. Mökkönen T. Studies on Stone Age Housepits in Fennoscandia (4000-2000 CalBC). Changes in Ground Plan, Site Location, and Degree of Sedentism. [Dissertation]. [Helsinki]: Helsinki University; 2011.

16. Kriiska A, Lõugas L, Lõhmus M, Mannermaa K, Johanson K. New AMS dates from Estonian Stone Age Burial sites. Est J Archaeol. 2007;11:83–121.

17. Pospieszny Ł. Freshwater reservoir effect and the radiocarbon chronology of the cemetery in Ząbie, Poland. J Archaeol Sci. 2015 Jan;53:264–76.

18. Pospieszny Ł. Zwyczaje pogrzebowe społeczności kultury ceramiki sznurowej w Wielkopolsce i na Kujawach. Wydawn. Poznańskie sp. z o.o.; 2009. 158 p.

19. Szczepanek A, Belka Z, Jarosz P, Pospieszny Ł, Dopieralska J, Frei KM, et al. Understanding Final Neolithic communities in south-eastern Poland: New insights on diet and mobility from isotopic data. PloS One. 2018;13(12):e0207748.

20. Włodarczak P. Radiocarbon and Dendrochronological Dates of the Corded Ware Culture. Radiocarbon. 2009 Jan;51(2):737–49.

21. Lindström J. De dödas tempel: om arkeologi och jakten på stenålderns själ. Stockholm: Norstedt; 2011. 382 p.

22. Brønstedt J. Danmarks Oldtid 1. Stenalderen. København: Gyldendal; 1957. 408 p.

23. Damm C. The Danish Single Grave Culture—Ethnic Migration or Social Construction? J Dan Archaeol. 1991 Jan;10(1):199–204.

24. Edgren T. Studier över den snörkeramiska kulturens keramik i Finland. Helsingfors; 1970. 118 p. (Suomen muinaismuistoyhdistyksen aikakauskirja 72).

25. Forssander J-E. Die schwedische Bootaxtkultur und ihre kontinentaleuropäischen Voraussetzungen. [Lund]: Borelius; 1933. 253p.

26. Kristiansen K. Prehistoric Migrations—the Case of the Single Grave and Corded Ware Cultures. J Dan Archaeol. 1989 Jan;8(1):211–25.

27. Malmer MP. The Neolithic of south Sweden: TRB, GRK, and STR. Stockholm: Royal Swedish Academy of Letters, History and Antiquities : Almqvist & Wiksell International; 2002. 284 p.

28. Malmer MP. Stridsyxekulturen i Sverige och Norge. Lund: Liber; 1975. 181 p.

29. Vandkilde H. A Review of the Early Late Neolithic Period in Denmark: Practice, Identity and Connectivity. J Neolit Archaeol. 2005;Dec:1–51.

30. Müller S. De jydske enkeltgrave fra Stenalderen. In: Aarböger for nordisk oldkyndighed og historie. Copenhagen; 1898. p. 157–216.

31. Carlsson A. Tolkande arkeologi och svensk forntidshistoria: från stenålder till vikingatid. Stockholm: Stockholm University, Department of Archaeology and Classical Studies; 1998. 79 p. (Stockholm studies in archaeology 17).

32. von Hackwitz K. The Creation of Regions: An Alternative Approach to Swedish Middle Neolithic Boundaries and Cultures. Nor Archaeol Rev. 2012 Jun;45(1):52–75.

33. Larsson ÅM. Breaking and making bodies and pots: material and ritual practices in Sweden in the third millennium BC. Uppsala: Department of Archaeology and Ancient History, Uppsala University; 2009. 455 p. (Aun 40).

34. Larsson ÅM. The hand that makes the pot: Craft traditions in south Sweden in the third Millenium BC. In: Berg I, editor. Breaking the Mould: Challenging Past Pottery. Prehist Ceram Res Group Occas Pap. 2008;6:81–91. (BAR International Series 1861).

35. Gimbutas M. The Kurgan Wave #2 (3400-3200 B.C.) into Europe and the Following Transformation of Culture. J Indo-Eur Stud. 1980;8(3):273–317.

36. Lang V. Some aspects of the Corded Ware Culture east of the Baltic Sea. In: Julku K, Wiik K, editors. The roots of peoples and languages of Northern Eurasia. Turku: Societas Historiae Fenno-Ugricae; 1998. p. 84–104.

37. Alvarez Romero J. People of the Corded Ware Culture in the East Baltic Region, Lithuania. Tyraetia. 2010;IV [XIX](1):139–47.

38. Jones ER, Zarina G, Moiseyev V, Lightfoot E, Nigst PR, Manica A, et al. The Neolithic Transition in the Baltic Was Not Driven by Admixture with Early European Farmers. Curr Biol. 2017 Feb;27(4):576–82.

39. Mittnik A, Wang C-C, Pfrengle S, Daubaras M, Zariņa G, Hallgren F, et al. The genetic prehistory of the Baltic Sea region. Nat Commun. 2018 Dec;9(1):442.

40. Saag L, Varul L, Scheib CL, Stenderup J, Allentoft ME, Saag L, et al. Extensive Farming in Estonia Started through a Sex-Biased Migration from the Steppe. Curr Biol. 2017 Jul;27(14):2185-2193.e6.

41. Fernandes DM, Strapagiel D, Borówka P, Marciniak B, Żądzińska E, Sirak K, et al. A genomic Neolithic time transect of hunter-farmer admixture in central Poland. Sci Rep. 2018 Dec;8(1):14879.

42. Juras A, Chyleński M, Ehler E, Malmström H, Żurkiewicz D, Włodarczak P, et al. Mitochondrial genomes reveal an east to west cline of steppe ancestry in Corded Ware populations. Sci Rep. 2018 Dec;8(1):11603.

43. Eriksson G. Part-time farmers or hard-core sealers? Västerbjers studied by means of stable isotope analysis. J Anthropol Archaeol. 2004 Jun;23(2):135–62.

44. Storå J. Reading bones: Stone Age hunters and seals in the Baltic. Stockholm: Univ; 2001. 86 p. (Stockholm studies in archaeology 21).

45. Fornander E. Dietary diversity and moderate mobility – isotope evidence from Scanian Battle Axe Culture burials. J Nord Archaeol Sci. 2013;18:13–29.

46. Lindström J, Samuelsson C, Ytterberg N. Long distance distribution of battle axes in Northern Europe - some aspects of the contacts between Denmark, Sweden and Finland 2800-2003 BC. In: Uniting Sea - Stone Age Societies in the Baltic Sea Region. Uppsala: Uppsala University; 2003. p. 147–60. (OPIA 33).

47. Heyd V. Kossinna's smile. Antiquity. 2017;91(356):348–59.

48. Allentoft ME, Sikora M, Sjögren K-G, Rasmussen S, Rasmussen M, Stenderup J, et al. Population genomics of Bronze Age Eurasia. Nature. 2015 Jun;522(7555):167–72.

49. Haak W, Lazaridis I, Patterson N, Rohland N, Mallick S, Llamas B, et al. Massive migration from the steppe was a source for Indo-European languages in Europe. Nature. 2015 Jun;522(7555):207–11.

50. Olalde I, Brace S, Allentoft ME, Armit I, Kristiansen K, Booth T, et al. The Beaker phenomenon and the genomic transformation of northwest Europe. Nature. 2018 Mar;555(7695):190–6.

51. Lindahl A. Dubbelgraven från stenåldern vid Bergsvägen i Linköping. In: Östergötlands och Linköpings stads Museum. 1955. p. 5–28. (Meddelanden).

52. Lindahl A, Gejvall N-G. Båtyxegraven från Bergsvägen i Linköping: En vägledning från Länsmuseet i Linköping. Östergötlands och Linköpings stads museum, editor. Linköping: Länsmuseet; 1973. 31 p.

53. Kaelas L. Till dateringen av båtyxegraven från Linköping. Fornvännen. 1956;1956:196–207.

54. Malmer MP. Dateringen av båtyxegraven från Linköping. Fornvännen. 1957;1957:17–30.

55. Malmström H, Linderholm A, Skoglund P, Storå J, Sjödin P, Gilbert MTP, et al. Ancient mitochondrial DNA from the northern fringe of the Neolithic farming expansion in Europe sheds light on the dispersion process. Philos Trans R Soc Lond B Biol Sci. 2015 Jan;370(1660):20130373.

56. Pawlak P. Byłem we wnętrzu, wszystko obejrzałem. Obłaczkowo – od neolitycznych osadników po średniowieczną wieś. Fontes Archaeol Posnanienses. 2013;49:231–327.

57. Hausmann R. Das Steinzeitgrab von Karlowa bei Dorpat. – Sitzungsberichte der Gelehrten Estnischen Gesellschaft. Jurew-Dorpat; 1911. 60–66 p.

58. Jaanits L, Laul S, Lõugas V, Tõnisson E. Eesti esiajalugu. Tallin: Eesti Raamat; 1982. 464 p.

59. Lõugas L, Kriiska A, Maldre L. New dates for the Late Neolithic Corded Ware Culture burials and early husbandry in the East Baltic region. Archaeofauna. 2007;16:21–31.

60. Magnusson M. Three Late Neolithic graves in east Skåne. In Lund: Lunds universitets historiska museum; 1947. p. 150–6. (Meddelanden från Lunds universitets historiska museum IV).

61. Brothwell DR. Digging up bones: the excavation, treatment and study of human skeletal remains. 3. ed. London: British Museum (Natural History); 1981. 208 p.

62. Ubelaker DH. Human skeletal remains: excavation, analysis, interpretation. 2. ed. Washington, D.C: Taraxacum; 1989. 172 p. (Manuals on archeology 2).

63. Buikstra JE, Ubelaker DH. Standards for data collection from human skeletal remains. Fayetteville: Arkansas Archeological Survey; 1994. 266 p. (Arkansas Archaeological Survey. Research Series 44).

64. Sjögren K-G. "Mångfalldige uhrminnes grafvar- ": megalitgravar och samhälle i Västsverige. Göteborg Univ. Institutionen för arkeologi; 2003. 381 p. (Coast to coast books. GOTARC Series B).

65. Cullberg C. Megalitgraven i Rössberga: utgrävningsrapport. Stockholm: Riksantikvarieämbetet; 1963. 122 p.

66. Persson P, Sjögren K-G, editors. Falbygdens gånggrifter. D. 1: Undersökningar 1985-1998. Göteborg: Institutionen för arkeologi, Göteborg Univ; 2001. 369 p. (GOTARC 34).

67. Sjögren K-G. C14 chronology of Scandinavian megalithic tombs. In: Exploring time and matter in prehistoric monuments: absolute chronology and rare rocks in European megaliths. In: Scarre C, Garcia Sanjuan L, Wheatley D, editors. Proceedings of the 2nd EMSG meeting (Sevilla, Nov 2008). Sevilla: Junta de Andalucía; 2011.

68. Ahlström T. Det döda kollektivet. Bioantropologisk analys av skelettmaterialet från Rössbergagånggriften. In: Persson P, Sjögren K-G, editors. Falbygdens gånggrifter Undersökningar 1985-1998. Göteborg: Institutionen för arkeologi, Göteborgs Universitet; 2001. p. 301–62. (GOTARC Serie C).

69. Linderholm A. Migration in prehistory: DNA and stable isotope analyses of Swedish skeletal material. Stockholm: Department of Archaeology and Classical Studies, Stockholm University; 2008. 84 p. (Theses and papers in scientific archaeology 10).

70. Burenhult G. The grave-field at Ajvide, Eksta, Gotland. In: Burenhult G, editor. Remote sensing: applied techniques for the study of cultural resources and the localization, identification and documentation of sub-surface prehistoric remains in Swedish archaeology Vol 2, Archaeological investigations, remote sensing case studies and osteo-anthropological studies. Hässelby: Stockholm University; 2002. p. 31–167. (Theses and papers in North-European archaeology 13b).

71. Burenhult G, Bartholin TS, editors. Remote sensing: applied techniques for the study of cultural resources and the localization, identification and documentation of sub-surface prehistoric remains in Swedish archaeology. Vol. 1: Osteo-anthropological, economic, environmental and technical analyses. Tjörnarp: G. Burenhult; 1997. 194 p. (Theses and papers in North-European archaeology 13a).

72. Norderäng J. Ajvideboplatsen : rapport från arkeologisk undersökning 2009 av fornlämning nr. 171 på fastigheten Ajvide 2:1 i Eksta socken, Gotland [Internet]. 2010 [cited 2019 Jun 19]. Available from: http://urn.kb.se/resolve?urn=urn:nbn:se:hgo:diva-193

73. Österholm I. Jakobs/Ajvide: undersökningar på en gotländsk boplatsudde från stenåldern. Visby: Gotland University Press; 2008. 297 p. (Gotland University Press).

74. Österholm I. The Jakobs/Ajvide site at Eksta. In: Remote sensing Applied techniques for the study of cultural resources and the localization, identification and documentation of sub-surface prehistoric remains in Swedish archaeology Volume II: Archaeological investigations, remote sensing case studies and osteo-anthropological studies. Hässelby: Stockholm University; 2002. p. 17–30. (Thesis and papers in north-european archaeology 13b).

75. Österholm I. Bosättningsmönstret på Gotland under stenåldern: en analys av fysisk miljö, ekonomi och social struktur. Stockholm: Univ; 1989. 4 p. (Theses and papers in archeology. New series, A).

76. Norderäng J. 14C-dateringar från Ajvide. In: Österholm I, editor. Jakobs/Ajvide: Undersökningar på en Gotländsk boplatsudde från stenåldern. Visby: Gotland University Press; 2008. p. 269–97.

77. Wallin P, Martinsson-Wallin H. Collective spaces and material expressions: ritual practice and island identities in Neolithic Gotland. In: Nash G, Townsend A, editors. Decoding Neolithic Atlantic and Mediterranean Island Ritual [Internet]. Havertown, UNITED STATES: Oxbow Books, Limited; 2016 [cited 2019 Jun 19]. p. 1–15. Available from: http://ebookcentral.proquest.com/lib/uu/detail.action?docID=4456615

78. Günther T, Malmström H, Svensson EM, Omrak A, Sánchez-Quinto F, Kılınç GM, et al. Population genomics of Mesolithic Scandinavia: Investigating early postglacial migration routes and high-latitude adaptation. Barton N, editor. PLOS Biol. 2018 Jan;16(1):e2003703.

79. Malmström H, Gilbert MTP, Thomas MG, Brandström M, Storå J, Molnar P, et al. Ancient DNA Reveals Lack of Continuity between Neolithic Hunter-Gatherers and Contemporary Scandinavians. Curr Biol. 2009 Nov;19(20):1758–62.

80. Skoglund P, Malmström H, Omrak A, Raghavan M, Valdiosera C, Günther T, et al. Genomic Diversity and Admixture Differs for Stone-Age Scandinavian Foragers and Farmers. Science. 2014 May;344(6185):747–50.

81. Skoglund P, Malmström H, Raghavan M, Storå J, Hall P, Willerslev E, et al. Origins and Genetic Legacy of Neolithic Farmers and Hunter-Gatherers in Europe. Science. 2012 Apr;336(6080):466–9.

82. Molnar P. Anthropological analysis of the skeletal material from Ajvide, Eksta sn., Gotland. In: Burenhult G, editor. Remote sensing Applied techniques for the study of cultural resources and the localization, identification and documentation of sub-surface prehistoric remains in Swedish archaeology Volume II: Archaeological investigations, remote sensing case studies and osteo-anthropological studies. Hässelby: Stockholm University; 2002. p. 371–4. (Thesis and papers in north-european archaeology 13b).

83. Bronk Ramsey C. Bayesian Analysis of Radiocarbon Dates. Radiocarbon. 2009 Jan;51(1):337–60.

84. Reimer PJ, Bard E, Bayliss A, Beck JW, Blackwell PG, Ramsey CB, et al. IntCal13 and Marine13 Radiocarbon Age Calibration Curves 0–50,000 Years cal BP. Radiocarbon. 2013 Mar;55(4):1869–87.

85. Fraser M. People of the dolmens and stone cists: an archaeogenetic investigation of megalithic graves from the Neolithic period on Gotland. Uppsala: Institutionen för arkeologi och antik historia; 2018. 83 p. (Aun 47).

86. Fraser M, Sanchez-Quinto F, Evans J, Storå J, Götherström A, Wallin P, et al. New insights on cultural dualism and population structure in the Middle Neolithic Funnel Beaker culture on the island of Gotland. J Archaeol Sci Rep. 2018 Feb;17:325–34.

87. Sánchez-Quinto F, Malmström H, Fraser M, Girdland-Flink L, Svensson EM, Simões LG, et al. Megalithic tombs in western and northern Neolithic Europe were linked to a kindred society. Proc Natl Acad Sci U S A. 2019 May;116(19):9469–74.

88. Sjögren K-G, Price TD, Kristiansen K. Diet and Mobility in the Corded Ware of Central Europe. PloS One. 2016;11(5):e0155083.

89. Eriksson G, Linderholm A, Fornander E, Kanstrup M, Schoultz P, Olofsson H, et al. Same island, different diet: Cultural evolution of food practice on Öland, Sweden, from the Mesolithic to the Roman Period. J Anthropol Archaeol. 2008 Dec;27(4):520–43.

90. Eriksson G, Frei KM, Howcroft R, Gummesson S, Molin F, Lidén K, et al. Diet and mobility among Mesolithic hunter-gatherers in Motala (Sweden) - The isotope perspective. J Archaeol Sci Rep. 2018 Feb;17:904–18.

91. Schlebusch CM, Malmström H, Günther T, Sjödin P, Coutinho A, Edlund H, et al. Southern African ancient genomes estimate modern human divergence to 350,000 to 260,000 years ago. Science. 2017 Nov;358(6363):652–5.

92. Yang DY, Eng B, Waye JS, Dudar JC, Saunders SR. Technical note: improved DNA extraction from ancient bones using silica-based spin columns. Am J Phys Anthropol. 1998 Apr;105(4):539–43.

93. Malmström H, Svensson EM, Gilbert MTP, Willerslev E, Götherström A, Holmlund G. More on Contamination: The Use of Asymmetric Molecular Behavior to Identify Authentic Ancient Human DNA. Mol Biol Evol. 2007 Jan;24(4):998–1004.

94. Dabney J, Knapp M, Glocke I, Gansauge M-T, Weihmann A, Nickel B, et al. Complete mitochondrial genome sequence of a Middle Pleistocene cave bear reconstructed from ultrashort DNA fragments. Proc Natl Acad Sci. 2013 Sep;110(39):15758–63.

95. Meyer M, Kircher M. Illumina Sequencing Library Preparation for Highly Multiplexed Target Capture and Sequencing. Cold Spring Harb Protoc. 2010 Jun;2010(6):pdb.prot5448-pdb.prot5448.

96. Günther T, Valdiosera C, Malmström H, Ureña I, Rodriguez-Varela R, Sverrisdóttir ÓO, et al. Ancient genomes link early farmers from Atapuerca in Spain to modern-day Basques. Proc Natl Acad Sci. 2015 Sep;112(38):11917–22.

97. Gansauge M-T, Meyer M. Single-stranded DNA library preparation for the sequencing of ancient or damaged DNA. Nat Protoc. 2013 Apr;8(4):737–48.

98. Kircher M. Analysis of High-Throughput Ancient DNA Sequencing Data. In: Shapiro B, Hofreiter M, editors. Ancient DNA [Internet]. Totowa, NJ: Humana Press; 2012 [cited 2019 Jun 11]. p. 197–228. Available from: http://link.springer.com/10.1007/978-1-61779-516-9_23

99. Li H, Handsaker B, Wysoker A, Fennell T, Ruan J, Homer N, et al. The Sequence Alignment/Map format and SAMtools. Bioinformatics. 2009 Aug;25(16):2078–9.

100. Skoglund P, Storå J, Götherström A, Jakobsson M. Accurate sex identification of ancient human remains using DNA shotgun sequencing. J Archaeol Sci. 2013 Dec;40(12):4477–82.

101. Sawyer S, Krause J, Guschanski K, Savolainen V, Pääbo S. Temporal Patterns of Nucleotide Misincorporations and DNA Fragmentation in Ancient DNA. Lalueza-Fox C, editor. PLoS ONE. 2012 Mar;7(3):e34131.

102. Green RE, Malaspinas A-S, Krause J, Briggs AW, Johnson PLF, Uhler C, et al. A Complete Neandertal Mitochondrial Genome Sequence Determined by High-Throughput Sequencing. Cell. 2008 Aug;134(3):416–26.

103. Rasmussen M, Guo X, Wang Y, Lohmueller KE, Rasmussen S, Albrechtsen A, et al. An Aboriginal Australian Genome Reveals Separate Human Dispersals into Asia. Science. 2011 Oct;334(6052):94–8.

104. Korneliussen TS, Albrechtsen A, Nielsen R. ANGSD: Analysis of Next Generation Sequencing Data. BMC Bioinformatics. 2014 Dec;15(1):356.

105. Vianello D, Sevini F, Castellani G, Lomartire L, Capri M, Franceschi C. HAPLOFIND: A New Method for High-Throughput mtDNA Haplogroup Assignment. Hum Mutat. 2013 Sep;34(9):1189–94.

106. van Oven M, Kayser M. Updated comprehensive phylogenetic tree of global human mitochondrial DNA variation. Hum Mutat. 2009 Feb;30(2):E386–94.

107. Behar DM, van Oven M, Rosset S, Metspalu M, Loogväli E-L, Silva NM, et al. A “Copernican” Reassessment of the Human Mitochondrial DNA Tree from its Root. Am J Hum Genet. 2012 Apr;90(4):675–84.

108. van Oven M, Van Geystelen A, Kayser M, Decorte R, Larmuseau MH. Seeing the Wood for the Trees: A Minimal Reference Phylogeny for the Human Y Chromosome. Hum Mutat. 2014 Feb;35(2):187–91.

109. Enattah NS, Sahi T, Savilahti E, Terwilliger JD, Peltonen L, Järvelä I. Identification of a variant associated with adult-type hypolactasia. Nat Genet. 2002 Feb;30(2):233–7.

110. Chaitanya L, Breslin K, Zuñiga S, Wirken L, Pośpiech E, Kukla-Bartoszek M, et al. The HIrisPlex-S system for eye, hair and skin colour prediction from DNA: Introduction and forensic developmental validation. Forensic Sci Int Genet. 2018 Jul;35:123–35.

111. Keller A, Graefen A, Ball M, Matzas M, Boisguerin V, Maixner F, et al. New insights into the Tyrolean Iceman's origin and phenotype as inferred by whole-genome sequencing. Nat Commun. 2012 Jan;3(1):698.

112. Fu Q, Li H, Moorjani P, Jay F, Slepchenko SM, Bondarev AA, et al. Genome sequence of a 45,000-year-old modern human from western Siberia. Nature. 2014 Oct;514(7523):445–9.

113. Gamba C, Jones ER, Teasdale MD, McLaughlin RL, Gonzalez-Fortes G, Mattiangeli V, et al. Genome flux and stasis in a five millennium transect of European prehistory. Nat Commun. 2014 Dec;5(1):5257.

114. Lazaridis I, Patterson N, Mittnik A, Renaud G, Mallick S, Kirsanow K, et al. Ancient human genomes suggest three ancestral populations for present-day Europeans. Nature. 2014 Sep;513(7518):409–13.

115. Seguin-Orlando A, Korneliussen TS, Sikora M, Malaspinas A-S, Manica A, Moltke I, et al. Genomic structure in Europeans dating back at least 36,200 years. Science. 2014 Nov 28;346(6213):1113–8.

116. Raghavan M, Skoglund P, Graf KE, Metspalu M, Albrechtsen A, Moltke I, et al. Upper Palaeolithic Siberian genome reveals dual ancestry of Native Americans. Nature. 2014 Jan;505(7481):87–91.

117. Cassidy LM, Martiniano R, Murphy EM, Teasdale MD, Mallory J, Hartwell B, et al. Neolithic and Bronze Age migration to Ireland and establishment of the insular Atlantic genome. Proc Natl Acad Sci. 2016 Jan;113(2):368–73.

118. Jones ER, Gonzalez-Fortes G, Connell S, Siska V, Eriksson A, Martiniano R, et al. Upper Palaeolithic genomes reveal deep roots of modern Eurasians. Nat Commun. 2015 Dec;6(1):8912.

119. Mathieson I, Lazaridis I, Rohland N, Mallick S, Patterson N, Roodenberg SA, et al. Genome-wide patterns of selection in 230 ancient Eurasians. Nature. 2015 Dec;528(7583):499–503.

120. Olalde I, Schroeder H, Sandoval-Velasco M, Vinner L, Lobón I, Ramirez O, et al. A Common Genetic Origin for Early Farmers from Mediterranean Cardial and Central European LBK Cultures. Mol Biol Evol. 2015 Sep;32(12):3132–42.

121. Omrak A, Günther T, Valdiosera C, Svensson EM, Malmström H, Kiesewetter H, et al. Genomic Evidence Establishes Anatolia as the Source of the European Neolithic Gene Pool. Curr Biol. 2016 Jan;26(2):270–5.

122. Mathieson I, Alpaslan-Roodenberg S, Posth C, Szécsényi-Nagy A, Rohland N, Mallick S, et al. The genomic history of southeastern Europe. Nature. 2018 Mar;555(7695):197–203.

123. Mallick S, Li H, Lipson M, Mathieson I, Gymrek M, Racimo F, et al. The Simons Genome Diversity Project: 300 genomes from 142 diverse populations. Nature. 2016 Oct;538(7624):201–6.

124. Sánchez-Quinto F, Schroeder H, Ramirez O, Avila-Arcos MC, Pybus M, Olalde I, et al. Genomic affinities of two 7,000-year-old Iberian hunter-gatherers. Curr Biol CB. 2012 Aug;22(16):1494–9.

125. Lazaridis I, Patterson N, Mittnik A, Renaud G, Mallick S, Kirsanow K, et al. Ancient human genomes suggest three ancestral populations for present-day Europeans. Nature. 2014 Sep;513(7518):409–13.

126. Omrak A, Günther T, Valdiosera C, Svensson EM, Malmström H, Kiesewetter H, et al. Genomic Evidence Establishes Anatolia as the Source of the European Neolithic Gene Pool. Curr Biol. 2016 Jan;26(2):270–5.

127. Alexander DH, Novembre J, Lange K. Fast model-based estimation of ancestry in unrelated individuals. Genome Res. 2009 Sep;19(9):1655–64.

128. Patterson N, Moorjani P, Luo Y, Mallick S, Rohland N, Zhan Y, et al. Ancient Admixture in Human History. Genetics. 2012 Nov;192(3):1065–93.

129. Lazaridis I, Nadel D, Rollefson G, Merrett DC, Rohland N, Mallick S, et al. Genomic insights into the origin of farming in the ancient Near East. Nature. 2016 Aug;536(7617):419–24.

130. Skoglund P, Mallick S, Bortolini MC, Chennagiri N, Hünemeier T, Petzl-Erler ML, et al. Genetic evidence for two founding populations of the Americas. Nature. 2015 Sep;525(7567):104–8.

131. Günther T, Nettelblad C. The presence and impact of reference bias on population genomic studies of prehistoric human populations. PLoS Genet. 2019 Jul;15(7):e1008302.

132. Rasmussen S, Allentoft ME, Nielsen K, Orlando L, Sikora M, Sjögren K-G, et al. Early divergent strains of Yersinia pestis in Eurasia 5,000 years ago. Cell. 2015 Oct;163(3):571–82.

133. Monroy Kuhn JM, Jakobsson M, Günther T. Estimating genetic kin relationships in prehistoric populations. Calafell F, editor. PLOS ONE. 2018 Apr;13(4):e0195491.
